# Associations among vegetation cover, particulate matter, and cardiovascular health in urban environments: a path analysis

**DOI:** 10.3389/fpls.2025.1659005

**Published:** 2025-11-07

**Authors:** Chengkang Wang, Xuyang Sun, Yuchong Wang, Zherui Bai, Lina Kang, Biao Xu, Jun Jin, Jiajie Cao, Yajing Mao, Xuan Wei, Huilin Liang

**Affiliations:** 1 Department of Cardiology, Nanjing Drum Tower Hospital, the Affiliated Hospital of Nanjing University Medical School, Nanjing, China; 2 School of Landscape Architecture, Nanjing Forestry University, Nanjing, China; 3 Research Institute of Architecture, Southeast University, Nanjing, China

**Keywords:** urban ecology, ecosystem services, mediation analysis, comparative ecosystem function, stress mitigation

## Abstract

**Introduction:**

Understanding the complex associative pathways linking urban green spaces to resident health is crucial for sustainable urban development and public health.

**Methods:**

This study aimed to investigate the indirect associations between residential vegetation cover (VC) and cardiovascular health, exploring the sequential roles of particulate matter (PM) and key physiological biomarkers in a large patient cohort. Using partial least squares structural equation modeling on 32,667 patient records from Nanjing, China, we constructed a series of path models to analyze these relationships.

**Results:**

Our findings reveal a significant indirect association between residential VC and cardiovascular health outcomes. Specifically, our path analysis reveals that higher VC is linked to lower concentrations of PM, with PM10 (particles ≤10mm) emerging as the dominant intermediary over PM2.5. In turn, lower PM10 levels are associated with healthier metabolic profiles—particularly lower total cholesterol and blood glucose levels —which were subsequently linked to better cardiovascular outcomes. Notably, total cholesterol was a key factor for reduced hospitalization frequency, while blood glucose was more strongly associated with lower incidence of heart failure. Among various vegetation metrics, Leaf Area Index demonstrated the strongest association within these pathways.

**Discussion:**

Our analysis provides evidence for a specific environmental health pathway (Vegetation → PM10 → Metabolic Biomarkers → Cardiovascular Outcomes) and highlights that vegetation quality, particularly Leaf Area Index, is a key factor. These findings offer valuable insights for urban planners and public health officials aiming to design healthier cities by leveraging the air-purifying benefits of urban green spaces.

## 1 Introduction

Cardiovascular diseases (CVDs) encompass a range of conditions affecting the heart and blood vessels and are the leading cause of mortality worldwide (Colantonio and Muntner, 2019; Li et al., 2023). With increasing prevalence, earlier onset, numerous complications, and high morbidity, mortality, disability, and recurrence rates, CVDs represent a significant global public health challenge (Joseph et al., 2017; Roth et al., 2020). Concurrently, substantial evidence demonstrates that urban natural environments, particularly green spaces, play a crucial role in improving human physiological health through various pathways (Liang et al., 2024c; Liang et al., 2024d). These natural elements are indispensable for sustainable urban development and enhancing the wellbeing of residents (Liang et al., 2024a; Liang et al., 2024b). Notably, several large-scale epidemiological studies have consistently revealed a robust negative correlation between the level of greenery in residential areas and CVD risk (Wang et al., 2019; Liu et al., 2022; Hajna et al., 2023). Residents in urban areas with higher vegetation cover (VC) exhibit a lower incidence and mortality rates of CVDs (Group et al., 2024), underscoring the potential of urban green spaces to promote cardiovascular health.

Extensive evidence demonstrates that urban green spaces contribute to cardiovascular health through multiple pathways. These natural environments provide opportunities for physical activity, stress reduction, and improved air quality by filtering harmful pollutants (Diener and Mudu, 2021; Jia et al., 2021; Qiu et al., 2021; Remme et al., 2021). Additionally, green spaces mitigate noise pollution (Dzhambov and Dimitrova, 2014; Browning et al., 2022), alleviate urban heat island effects (Sun and Chen, 2017; van den Bosch and Sang, 2017), and enhance immune function through exposure to beneficial microbes (Rook, 2013; Sandifer et al., 2015). Recognizing these benefits, healthcare professionals increasingly offer so-called green prescriptions, encouraging patients to engage with nature as part of lifestyle interventions (Shanahan et al., 2019; Adewuyi et al., 2023). This approach positions urban greenery as a promising strategy for CVD prevention. Consequently, there is a growing need to integrate epidemiological and urban environmental research to explore the complex relationships and potential pathways linking urban natural environments to cardiovascular health. Such understanding is crucial for incorporating health considerations into urban green space planning, guiding urban development strategies, and maximizing the potential of urban natural environments to promote cardiovascular wellbeing. While existing research consistently demonstrates a protective association between residential greenness and cardiovascular health (Yitshak-Sade et al., 2017; Jennings et al., 2019; Wang et al., 2019; Donovan et al., 2022; Hajna et al., 2023), these studies have predominantly relied on correlation or regression analyses. Consequently, the complex, multi-step pathways through which green spaces are linked to these cardiovascular benefits remain insufficiently understood, representing a critical gap in the literature.

Recognizing the multifaceted pathways through which green spaces influence population health, recent studies have incorporated mediating factors such as physical activity (Richardson et al., 2013; Jia et al., 2018), air temperature (Shen and Lung, 2016), and traffic noise (Orioli et al., 2019) to better understand the pathways underlying the relationship between green spaces and cardiovascular health. Notably, air pollution has emerged as a prominent mediator, with research demonstrating the ecological benefits of green spaces for reducing air particulate matter (APM) (Diener and Mudu, 2021). For instance, cohort studies in China identified PM2.5 as a partial mediator in the beneficial association between green spaces and atherosclerosis (Hu et al., 2022; Li et al., 2022). Furthermore, a comprehensive literature review and meta-analysis confirmed that green spaces can promote cardiovascular health by reducing air pollution through ecosystem regulation services (Qiu et al., 2021). However, the association between air pollution and cardiovascular health is complex. It involves various physiological factors such as lipid abnormalities (Cesaroni et al., 2014), hypertension (Lei et al., 2022), hyperglycemia (Yang et al., 2018), and inflammatory responses (Burkart et al., 2022). Additionally, physiological health risks (PHRs) have been recognized as potential mediators, with studies revealing protective associations between residential greenness and allostatic load (Lai et al., 2024), and the mediating role of cardiometabolic disorders in the relationship between green spaces and CVD (Yang et al., 2020). Therefore, simultaneously considering air quality and PHR as mediating factors can offer a more comprehensive and scientifically robust approach to understanding the complex mechanisms linking green spaces to cardiovascular health outcomes (CHOs).

Current research on the relationship between green spaces and cardiovascular health predominantly focuses on single-factor mediation analyses. Even studies incorporating multiple potentially interrelated mediators often conduct independent analyses or examine parallel mediation effects. In doing so, they neglect the potential for chain-mediated interactions between these factors, that is, a sequential pathway where green space is associated with air quality, which in turn is associated with physiological risk, ultimately linking to health outcomes (Shen and Lung, 2016; Dzhambov et al., 2020; Dong et al., 2021). While investigations into serial mediators in the relationship between green spaces and health are limited, some studies have made notable contributions. For instance, one study demonstrated a partial chain-mediated effect of air pollutants (PM2.5, NO_2_) and perceived air pollution in the beneficial impact of urban street tree exposure on mental health (Wang et al., 2020). Another study of middle-aged and elderly Chinese found that the pathway between PM2.5 concentrations and physical activity partially mediated the association between residential greenness and hypertension in rural areas (Huang et al., 2021). These findings provide support and inspiration for further exploring the complex mechanisms underlying the impact of green spaces on physiological health. Therefore, this study aimed to address these gaps by: (1) Investigating the association between residential VC and CHOs in a large patient cohort from Nanjing, China; (2) Exploring the potential indirect pathways of association linking VC and CHOs via particulate matter (PM) and PHR biomarkers using structural equation modeling; and (3) Comparing the relative strength of association for different indicators of VC, PM, and PHR within these associative pathways.

Our study aimed to elucidate the pathways of association linking urban green space VC to the cardiovascular health of residents. Specifically, we explored the indirect associations between residential green space VC and CHOs through a sequential pathway involving air quality and PHR. Recognizing that single indicators may lead to bias, we employed a multi-indicator approach to comprehensively characterize each factor. This method facilitates a more in-depth exploration of the indirect pathways associating green spaces with cardiovascular health via air quality and PHR. By comparing the roles of different indicators within the path model, we aimed to clarify the relative strength of association for each. This research can provide a reference and evidence-based support for urban green space planning, cardiovascular disease prevention strategies, and patient recovery interventions. Furthermore, our findings can contribute to the development of more effective urban planning and public health policies that leverage the health benefits of urban green spaces.

## 2 Study site and sample

Nanjing, a major metropolis in eastern China, comprises 11 administrative districts covering a total area of 6,587.04 km^2^, with an urban built-up area of 868.28 km^2^. As of late 2023, Nanjing’s permanent resident population was 9.547 million. Over the past decade, the city has intensively implemented its Green Nanjing strategy to improve the urban park system and greenway network while strengthening ecological environmental construction and protection. By the end of 2022, the city’s forest coverage area exceeded 297 million mu, with a tree coverage rate of 31.96%, an urban green coverage rate of 44.96%, and a green space rate of 40.82% (Statistics, N.B.o, 2023). Nanjing faces significant challenges related to population aging and cardiovascular health. The policy Key Tasks for Healthy Nanjing Construction in 2023 explicitly outlines strategic guidelines for the prevention and treatment of cardiovascular and cerebrovascular diseases, underscoring the practical and strategic importance of research in this region.

We conducted a retrospective analysis of electronic medical records from 81,306 patients with cardiovascular department hospitalization records at all campuses of Nanjing Drum Tower Hospital (including the Gulou and Jiangbei campuses) from January 2013 to December 2023. After screening, 32,667 patient records from December 2014 to December 2023 were selected for the study, based on criteria including detailed and accurate address information, complete key physiological indicator test data, and residence within Nanjing city limits. For each selected patient, environmental exposures (i.e., VC and air PM) were assessed within a 1000-meter buffer zone around their registered residence. The participant selection process is detailed in **Supplementary Figure S1**.

## 3 Materials and methods

### 3.1 Data collection and pretreatment

To investigate the complex pathways of association by which VC in green spaces is linked with cardiovascular health, involving the intermediate roles of air quality and various PHRs, we employed four latent variables: VC, APM, PHR, and CHOs. Each latent variable encompassed multiple observed variables (**Table 1**). The bivariate correlations between all observed variables are presented in **Supplementary Figure S2** in the **Supplementary Material**. For a comprehensive assessment of VC around patients’ residences, six vegetation indices were used as observed variables for VC. Given the known effects of VC on air quality, particularly APM (Diener and Mudu, 2021), PM2.5 and PM10 were selected as observed variables for APM. Based on PHRs leading to adverse cardiovascular outcomes (Hu, 2023), and to avoid potential temporal confounding from acute-phase reactants, we excluded inflammatory markers. Consequently, three second-level latent variables were chosen for PHR: blood lipid risk (BLR), blood glucose risk (BGR), and blood pressure risk (BPR). Corresponding biomarkers were selected as observed variables for these risks (**Table 1**). For CHOs, hospitalization utilization (HU) and cardiovascular disease diagnosis (CVDD) were chosen as latent variables. Data for these variables were collected from various sources, including satellite imagery for vegetation indices, air quality monitoring networks for APM, and electronic medical records for PHR and CHOs. For each patient, environmental exposure data (VC and PM) were calculated as annual averages for the calendar year preceding their hospital admission date. Appendix A provides the rationale for indicator selection, detailed data collection procedures, and data processing methods.

**Table 1:** Variables of chain mediation models in this study.

Latent variables	Observed variables	Calculation methods and units
Independent variable		
VC	Normalized difference vegetation index (NDVI)	(B5 - B4)/(B5 + B4) (Pettorelli, 2013)
	Enhanced vegetation index (EVI)	2.5*(B5-B4)/(B5 + 6*B4 -7.5*B2 + 1) (Liu and Huete, 1995)
	Leaf Area Index (LAI)	8.278 * NDVI - 0.2459 (Allegrini et al., 2006)
	Forest vegetation coverage (FVC)	(NDVI - NDVIs)/(NDVIv -NDVIs) (Song et al., 2017)
	Soil adjusted Vegetation Index (SAVI)	(1 + L) * (B5 - B4)/(B5 + B4 + L) (Huete, 1988)
	Difference vegetation index (DVI)	B5 - B4 (Yan et al., 2022)
Mediating variable		
APM	PM 2.5 concentration	annual average, μg/m^3^
	PM 10 concentration	annual average, μg/m^3^
PHR		
Blood lipid risk (BLR)	Total cholesterol (TC)	mmol/L
	High density lipoprotein cholesterol (HDL-C)	mmol/L
Blood glucose risk (BGR)	Blood glucose (GLU)	mmol/L
Blood pressure risk (BPR)	Systolic blood pressure (SBP)	mmHg
Dependent variable		
CHO		
Hospital utilization (HU)	Times of hospitalizations (TH)	Times
	Days of hospitalizations (DH)	Days
Cardiovascular disease diagnosis (CVDD)	Diagnosed chronic ischemic heart disease (CIHD)	Categorical variable
	Diagnosed heart failure (HF)	Categorical variable

VC, Vegetation cover; APM, Airborne particulate matter; PHR, Physiological health risk; CHO, Cardiovascular health outcome.

### 3.2 Chain mediation models

**Figure 1:**
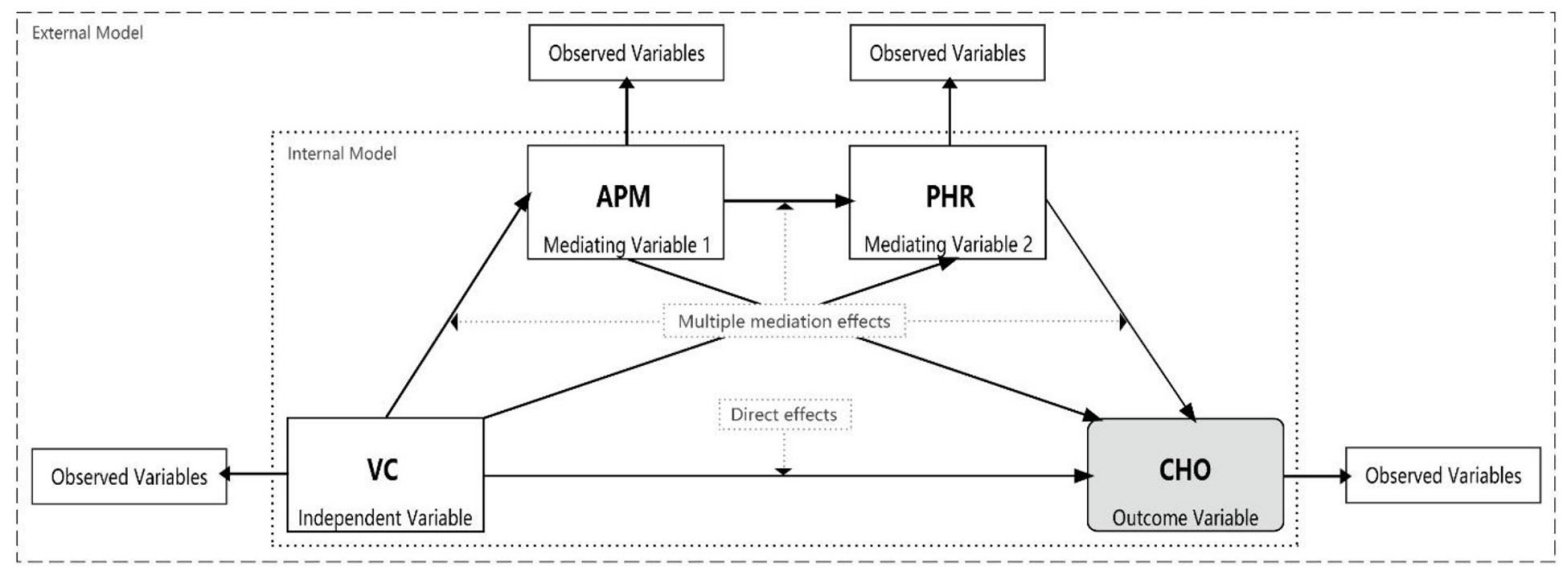
Research model framework. VC, Vegetation cover; APM, Physiological health risk; PHR, Airborne particulate matter; CHO, cardiovascular health outcome.

To explore the complex pathways of association linking VC to CHOs, we employed a pre-specified, four-stage analytical strategy using a series of path models. This hierarchical approach was designed to move progressively from broad, composite constructs to more specific, granular indicators, allowing for a systematic exploration of the associative pathways. VC was treated as the exogenous variable, CHOs as the final endogenous variables, with APM and PHR positioned as intermediate variables in the pathway. The observed variables for each latent variable are presented in **Table 1**. Each of the four model groups (Model Groups 1-4) was analyzed using partial least squares structural equation models (PLS-SEMs) (**Figure 1**).

#### 3.2.1 Model group 1: overall associative model with composite latent variables

This group of models constructs two chain mediation effect models, Model 1a and Model 1b, respectively using HU and CVDD as dependent variables. These models analyze the association of VC with HU and CVDD, exploring the indirect pathway via APM and PHR. The external models are as follows (Equations 1–5):

(1) $$\xi_{VC}=\sum \left(\lambda_{VC_{i}}*x_{VC_{i}}\right)+\delta_{VC},\;\;for\;VC_{i}\in \left\{NDVI,\;EVI,\;SAVI,\;DVI,\;FVC,\;LAI\right\}$$

(2) $$\eta_{APM}=\sum \left(\lambda_{APM_{j}}*x_{APM_{j}}\right)+\epsilon_{APM},\;\;for\;APM_{j}\in \left\{PM2.5,\;PM10\right\}$$

(3) $$\eta_{PHR}=\sum \left(\lambda_{BM_{k}}*x_{BM_{k}}\right)+\epsilon_{PHR},\;\;for\;BM_{k}\in \left\{TC,\;HDL-C,\;GLU,\;SBP,\;DBP\right\}$$

(4) $$\eta_{HU}=\sum \left(\lambda_{HU_{l}}*x_{HU_{l}}\right)+\epsilon_{HU},\;\;for\;HU_{l}\in \left\{TH,\;DH\right\}$$

(5) $$\eta_{CVDD}=\sum \left(\lambda_{CVDD_{l}}*x_{CVDD_{l}}\right)+\epsilon_{CVDD},\;\;for\;CVDD_{l}\in \left\{CIHD,\;HF\right\}$$

where ξ and η represent exogenous and endogenous latent variables, respectively, *x* represents observed variables, λ represents factor loadings of observed variables, δ represents measurement errors of observed variables for exogenous latent variables, and ϵ represents measurement errors of observed variables for endogenous latent variables.

As each latent variable in this model group is a single factor, the constructed models are single-factor models. Their internal models are as follows (Equations 6–9):

(6) $$\eta_{APM}=\gamma_{VC}*\xi_{VC}+\zeta_{APM}$$

(7) $$\eta_{PHR}=\beta_{APM\to PHR}*\eta_{APM}+\gamma_{VC\to PHR}*\xi_{VC}+\zeta_{PHR}$$

(8) $$\eta_{HU}=\beta_{PHR\to HU}*\eta_{PHR}+\beta_{APM\to HU}*\eta_{APM}+\gamma_{VC\to HU}*\xi_{VC}+\zeta_{HU}$$

(9) $$\eta_{CVDD}=\beta_{PHR\to CVDD}*\eta_{PHR}+\beta_{APM\to CVDD}*\eta_{APM}+\gamma_{VC\to CVDD}*\xi_{VC}+\zeta_{CVDD}$$

where γ represents path coefficients from exogenous latent variables to endogenous latent variables, indicating the direct influence of exogenous latent variables on endogenous latent variables; β represents path coefficients between endogenous latent variables, indicating the association between endogenous latent variables; and ζ represents prediction errors of endogenous latent variables.

#### 3.2.2 Model group 2: deconstructing PHR pathways

This group of models builds upon Model Group 1 by further subdividing the CHO variables (HU and CVDD) into specific indicators: times of hospitalization (TH), days of hospitalization (DH), diagnosed chronic ischemic heart disease (CIHD), and diagnosed heart failure (HF). Four chain mediation effect models (Models 2a, 2b, 2c, and 2d) are constructed with these four variables as dependent variables. Additionally, PHR is subdivided into latent variables BLR, BGR, and BPR to analyze their roles as intermediate variables in the pathway linking VC, APM, and each CHO (TH, DH, CIHD, and HF). The external models for $\xi_{VC}$ and $\eta_{APM}$ are the same as for Models (1-1) and (1-2) in Model Group 1. The remaining external models are as follows (Equations 10–13):

(10) $$\eta_{BLR}=\sum \left(\lambda_{BLR_{k}}*x_{BLR_{k}}\right)+\epsilon_{BLR},\;\;for\;BLR_{k}\in \left\{TC,\;HDL-C\right\}$$

(11) $$\eta_{BGR}=\lambda_{BGR}*x_{GLU}+\epsilon_{BGR}$$

(12) $$\eta_{BPR}=\sum \left(\lambda_{BPR_{k}}*x_{BPR_{k}}\right)+\epsilon_{BPR},\;\;for\;BPR_{k}\in \left\{SBP,\;DBP\right\}$$

(13) $$\eta_{CHO_{l}}=\lambda_{CHO_{l}}*x_{CHO_{l}}+\epsilon_{CHO_{l}},\;for\;CHO_{l}\in \left\{TH,\;DH,\;CIHD,\;HF\right\}$$

In this model group, $\xi_{VC}$ and $\eta_{APM}$ are single factors. Their internal model is the same as for Model (1-6) in Model Group 1. The internal model for $\eta_{PHRi}$ is as follows (Equations 14–16):

(14) $$\begin{array}{c} \eta_{PHR_{k}}=\beta_{APM\to PHR_{k}}*\eta_{APM}+\gamma_{VC\to PHR_{k}}*\xi_{VC}+\zeta_{PHR_{k}},\;\;\\ for\;PHR_{k}\in \left\{BLR,\;BGR,\;BPR\right\} \end{array}$$

As there may be interaction effects between the different PHR variables, we construct a comprehensive-effect model to fully reflect the combined influence of all PHR variables on CHO. The internal model for $\eta_{CHOi}$ is as follows:

(15) $$\eta_{CHO_{l}}=\sum \left(\beta_{PHR_{k}\to CHO_{l}}*\eta_{PHR_{k}}\right)+\beta_{APM\to CHO_{l}}*\eta_{APM}+\gamma_{VC\to CHO_{l}}*\xi_{VC}+\zeta_{CHO_{l}}$$

If the comprehensive-effect model encounters issues such as multicollinearity, we consider constructing a single-factor model. The internal model for $\eta_{CHOi}$ in this case would be

(16) $$\eta_{CHO_{l}}=\beta_{PHR_{k}\to CHO_{l}}*\eta_{PHR_{k}}+\beta_{APM\to CHO_{l}}*\eta_{APM}+\gamma_{VC\to CHO_{l}}*\xi_{VC}+\zeta_{CHO_{l}}$$

#### 3.2.3 Model group 3: deconstructing PM and biomarker pathways

The third stage of our analytical strategy was designed to simultaneously deconstruct both the APM and PHR constructs to identify the most salient pathways at a granular level. This group consists of four path models (Models 3a, 3b, 3c, and 3d), one for each CHO. Based on the findings from Model Group 2, which highlighted the low internal consistency of the BLR construct and the primary roles of lipid and glucose pathways, our pre-specified plan was to disaggregate the PHR constructs directly into their constituent, clinically-measured biomarkers: total cholesterol (TC), high-density lipoprotein cholesterol (HDL-C), and blood glucose (GLU). Concurrently, the APM construct was disaggregated into PM2.5 and PM10 to assess their distinct roles. This integrated approach allows for a direct, competitive comparison of the pathways running through different PM sizes to specific biomarkers within a single, comprehensive model.

The external models for $\xi_{VC}$, $\eta_{TH}$, $\eta_{DH}$, $\eta_{CIHO}$, and $\eta_{HF}$ are the same as in previous groups. The remaining external models are as follows (Equations 17, 18):

(17) $$\eta_{APM_{j}}=\lambda_{APM_{j}}*x_{APM_{j}}+\epsilon_{APM_{j}},\;\;for\;APM_{j}\in \left\{PM2.5,\;PM10\right\}$$

(18) $$\eta_{BM_{k}}=\lambda_{BM_{k}}*x_{BM_{k}}+\epsilon_{BM_{k}},\;for\;BM_{k}\in \left\{TC,\;HDL-C,\;GLU\right\}$$

where $\eta_{APMi}$ represents the individual PM variables and $\eta_{BMj}$ represents the individual biomarker variables.

To comprehensively reflect the combined influence of all variables, our primary analytical approach was to construct a comprehensive-effect model. The internal models for this approach are as follows (Equations 19–21):

(19) $$\eta_{APM_{j}}=\gamma_{VC\to APM_{j}}*\xi_{VC}+\zeta_{APM_{j}}$$

(20) $$\eta_{BM_{k}}=\sum \left(\beta_{APM_{j}\to BM_{k}}*\eta_{APM_{j}}\right)+\gamma_{VC\to BM_{k}}*\xi_{VC}+\zeta_{BM_{k}}$$

(21) $$\eta_{CHO_{l}}=\sum \left(\beta_{BM_{k}\to CHO_{l}}*\eta_{BM_{k}}\right)+\sum \left(\beta_{APM_{j}\to CHO_{l}}*\eta_{APM_{j}}\right)+\gamma_{VC\to CHO_{l}}*\xi_{VC}+\zeta_{CHO_{l}}$$

where $\eta_{CHOk}$ represents one of the four final health outcomes (TH, DH, CIHD, or HF).

Given the potential for multicollinearity between PM2.5 and PM10, our analytical plan specified that if the comprehensive-effect model showed evidence of multicollinearity (e.g., VIF > 5), we would proceed with single-factor models for the PM variables to ensure a clear interpretation. The internal models for this single-factor approach would be (Equations 22, 23):

(22) $$\eta_{BM_{k}}=\beta_{APM_{j}\to BM_{k}}*\eta_{APM_{j}}+\gamma_{VC\to BM_{k}}*\xi_{VC}+\zeta_{BM_{k}}$$

(23) $$\eta_{CHO_{l}}=\beta_{BM_{k}\to CHO_{l}}*\eta_{BM_{k}}+\beta_{APM_{j}\to CHO_{l}}*\eta_{APM_{j}}+\gamma_{VC\to CHO_{l}}*\xi_{VC}+\zeta_{CHO_{l}}$$

#### 3.2.4 Model group 4: comparing individual VC indicators

The final stage of our analysis, Model Group 4, was designed to investigate the relative importance of different vegetation characteristics. In this stage, the composite VC construct was disaggregated into its six individual indicators (NDVI, EVI, SAVI, DVI, FVC, and LAI). To construct the most salient and parsimonious path for this comparative analysis, our pre-specified plan was to utilize the single strongest APM indicator and the single most influential biomarker identified from the preceding stage (Model Group 3). This allowed for a direct comparison of the association strength of each individual VC metric within a consistent and statistically powerful pathway.

The external models for $\eta_{TH}$, $\eta_{DH}$, $\eta_{CIHO}$, and $\eta_{HF}$ are the same as for Model (2-4) in Model Group 2. The remaining external models are as follows (Equations 24–26):

(24) $$\xi_{VC_{i}}=\lambda_{VC_{i}}*x_{VC_{i}}+\delta_{VC_{i}},\;\;for\;VC_{i}\in \left\{NDVI,\;EVI,\;SAVI,\;DVI,\;FVC,\;LAI\right\}$$

(25) $$\eta_{APM_{max}}=\lambda_{APM_{max}}*x_{APM_{max}}+\epsilon_{APM_{max}}$$

(26) $$\eta_{BM_{max}}=\lambda_{BM_{max}}*x_{BM_{max}}+\epsilon_{BM_{max}}$$

where $\eta_{APMmax}$ represents the selected APM variable with the strongest association (determined from Model Group 3 results), $x_{APM_{max}}$ represents its observed variable, $\eta_{BM_{max}}$ represents the selected biomarker variable with the strongest association (determined from Model Group 3 results), and $x_{BMmax}$ represents its observed variable.

To comprehensively reflect the combined influence of all VC indicators on APM, our primary analytical approach was to construct a comprehensive-effect model. The internal models for this approach are as follows (Equations 27–29):

(27) $$\eta_{APM_{max}}=\sum \left(\gamma_{VC_{i}\to APM}*\xi_{VC_{i}}\right)+\zeta_{APM_{max}}$$

(28) $$\eta_{BM_{max}}=\beta_{APM\to BM}*\eta_{APM_{max}}+\sum \left(\gamma_{VC_{i}\to BM}*\xi_{VC_{i}}\right)+\zeta_{BM_{max}}$$

(29) $$\eta_{CHO_{l}}=\beta_{BM_{max}}*\eta_{BM_{max}}+\beta_{APM_{max}}*\eta_{APM_{max}}+\sum \left(\gamma_{VC_{i}\to CHO_{l}}*\xi_{VC_{i}}\right)+\zeta_{CHO_{l}}$$

However, given the high potential for multicollinearity among the individual VC indicators, our analytical plan specified that if the comprehensive-effect model showed evidence of multicollinearity (e.g., VIF > 5), we would proceed with single-factor models to ensure a clear and direct comparison of each indicator’s effect. The internal models for this single-factor approach are as follows (Equations 30–32):

(30) $$\eta_{APM_{max}}=\gamma_{VC_{i}\to APM}*\xi_{VC_{i}}+\zeta_{APM_{max}}$$

(31) $$\eta_{BM_{max}}=\beta_{APM\to BM}*\eta_{APM_{max}}+\gamma_{VC_{i}\to BM}*\xi_{VC_{i}}+\zeta_{BM_{max}}$$

(32) $$\eta_{CHO_{l}}=\beta_{BM\to CHO_{l}}*\eta_{BM_{max}}+\beta_{APM\to CHO_{l}}*\eta_{APM_{max}}+\gamma_{VC_{i}\to CHO_{l}}*\xi_{VC_{i}}+\zeta_{CHO_{l}}$$

These four groups of path models allow for the decomposition of the total association between VC and CHOs into direct and indirect pathways involving APM and PHR. The terms “direct effect” and “indirect effect” are used here to describe statistical paths within the model, not to imply causality. Each group progressively refines the analysis, from broad concepts to specific indicators. As the coefficients and effects in the models are standardized, the relative strengths of association across different variables and pathways can be directly compared. To test the statistical significance of relationships and effects in the models, we employed the bootstrap method, generating 5000 subsamples for each model. This method provides robust estimates of standard errors and confidence intervals for model parameters. We evaluated the validity and reliability of the external model using composite reliability (CR), Cronbach’s α, and the square root of the average variance extracted (AVE). These metrics assess the internal consistency and convergent validity of the constructs. The internal model’s collinearity was assessed using the variance inflation factor (VIF), which helps identify potential multicollinearity issues. The overall model fit was evaluated using the standardized root mean square residual (SRMR) and the normed fit index (NFI), which provide measures of the discrepancy between the observed and model-implied correlation matrices. All mediation effect models were analyzed using SmartPLS software (Version 4.1, SmartPLS GmbH, Germany).

## 4 Results

### 4.1 Overall associations among composite latent variables

The model evaluation results (**Supplementary Table S1**) indicate that the composite constructs for VC and APM have high reliability and validity. However, the overall PHR construct, now excluding inflammatory markers, still shows CR, AVE, and Cronbach’s α values below recommended thresholds, suggesting heterogeneity among the remaining physiological risks. The adjusted R² values for the final outcomes were 0.120 for HU (Model 1a) and 0.080 for CVDD (Model 1b), indicating modest explanatory power at this broad level.

**Figure 2:**
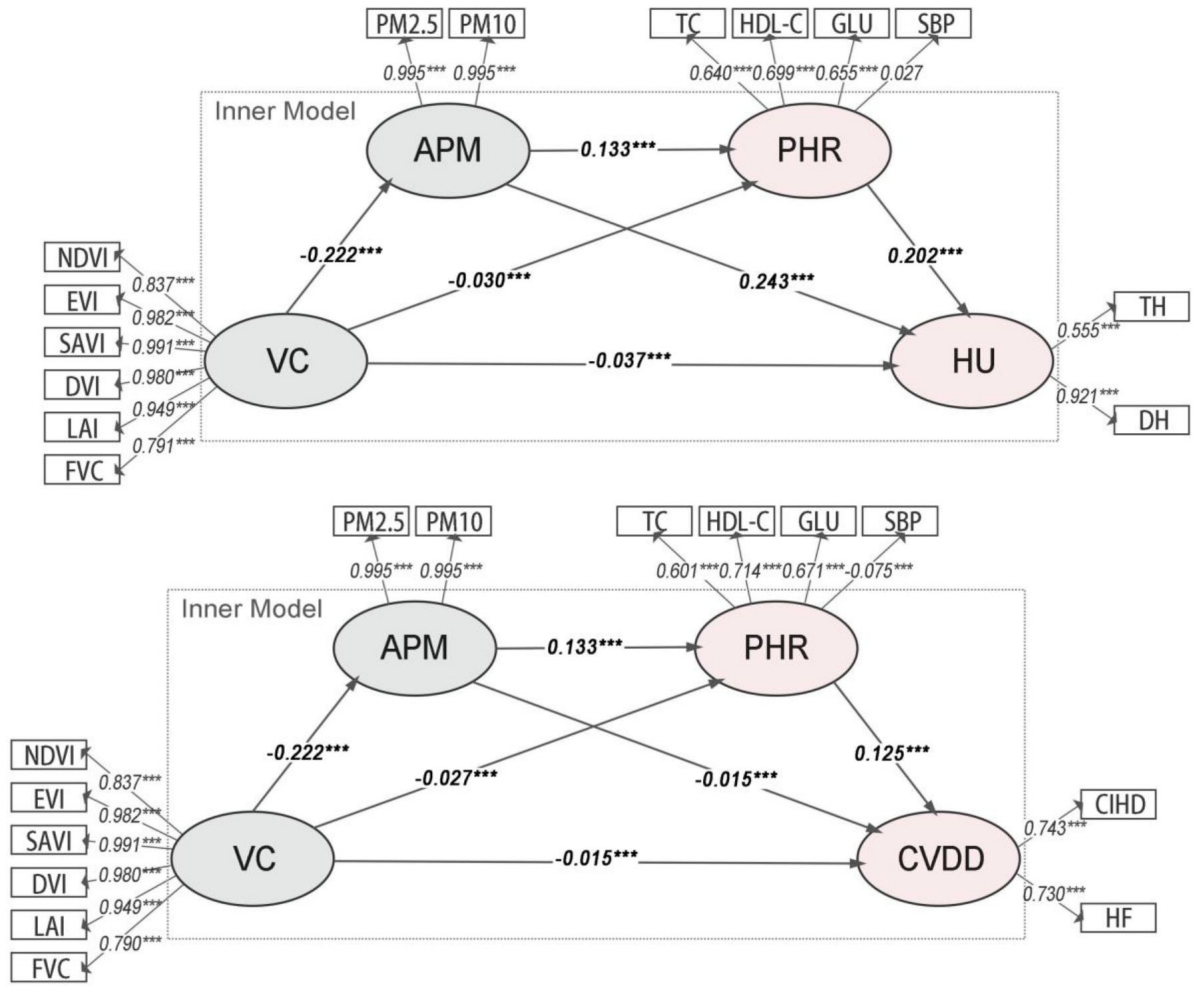
Path diagrams of the PLS-SEMs in model group 1. VC, Vegetation cover; PHR, Physiological health risk; APM, Airborne particulate matter; HU, Hospital utilization; CVDD, Cardiovascular disease diagnosis.

The path analysis results for Model Group 1 (**Figure 2**) show that all path coefficients are statistically significant. VC was significantly and negatively associated with APM (β = -0.222, p< 0.001). In turn, APM was positively associated with the composite PHR (β = 0.132 in Model 1a; β = 0.131 in Model 1b, p< 0.001). Finally, PHR was significantly associated with both HU (β = 0.315, p< 0.001) and CVDD (β = 0.245, p< 0.001).

The analysis of indirect associations (**Table 2**) reveals that all pathways are statistically significant. For HU (Model 1a), the primary indirect pathway is VC→APM→HU (Specific Indirect Effect = -0.05352, p< 0.001). For CVDD (Model 1b), a notable inconsistent association was observed: while the total association was negative (Total Effect = -0.01866, p< 0.001), the specific indirect path through APM alone (VC→APM→CVDD) was positive (Specific Indirect Effect = 0.00333, p< 0.001). This statistical phenomenon, potentially indicative of a suppressor effect, suggests complex underlying relationships that warrant further disaggregation in subsequent models.

**Table 2:** Indirect associations in the PLS-SEMs of model group 1.

Model pathway	Total effect	Direct effect	Specific indirect effect
Model 1a (VC→APM→PHR→HU)			
VC→APM→HU	-0.10283^***^	-0.03681^***^	-0.05352^***^
VC→PHR→HU	-0.10283^***^	-0.03681^***^	-0.00629^***^
VC→APM→PHR→HU	-0.10283^***^	-0.03681^***^	-0.00741^***^
Model 1b (VC→APM→PHR→CVDD)			
VC→APM→CVDD	-0.01866^***^	-0.01496^***^	0.00333^***^
VC→PHR→CVDD	-0.01866^***^	-0.01496^***^	-0.00334^***^
VC→APM→PHR→CVDD	-0.01866^***^	-0.01496^***^	-0.00369^***^

VC, Vegetation cover; PHR, Physiological health risk; APM, Airborne particulate matter; HU, Hospital utilization; CVDD, Cardiovascular disease diagnosis.

^*^p< 0.05; ^**^p< 0.01; ^***^p< 0.001.

### 4.2 Associations involving disaggregated physiological health risks

In Model Group 2, the PHR construct was disaggregated into BLR, BGR, and BPR. Model evaluation (**Supplementary Table S2**) showed that while VC and APM remained robust, the BLR construct had a low Cronbach’s α (0.463). This low internal consistency indicates that TC and HDL-C do not form a reliable single construct, methodologically necessitating the disaggregation of BLR into its constituent biomarkers in the subsequent analytical stage.

**Figure 3:**
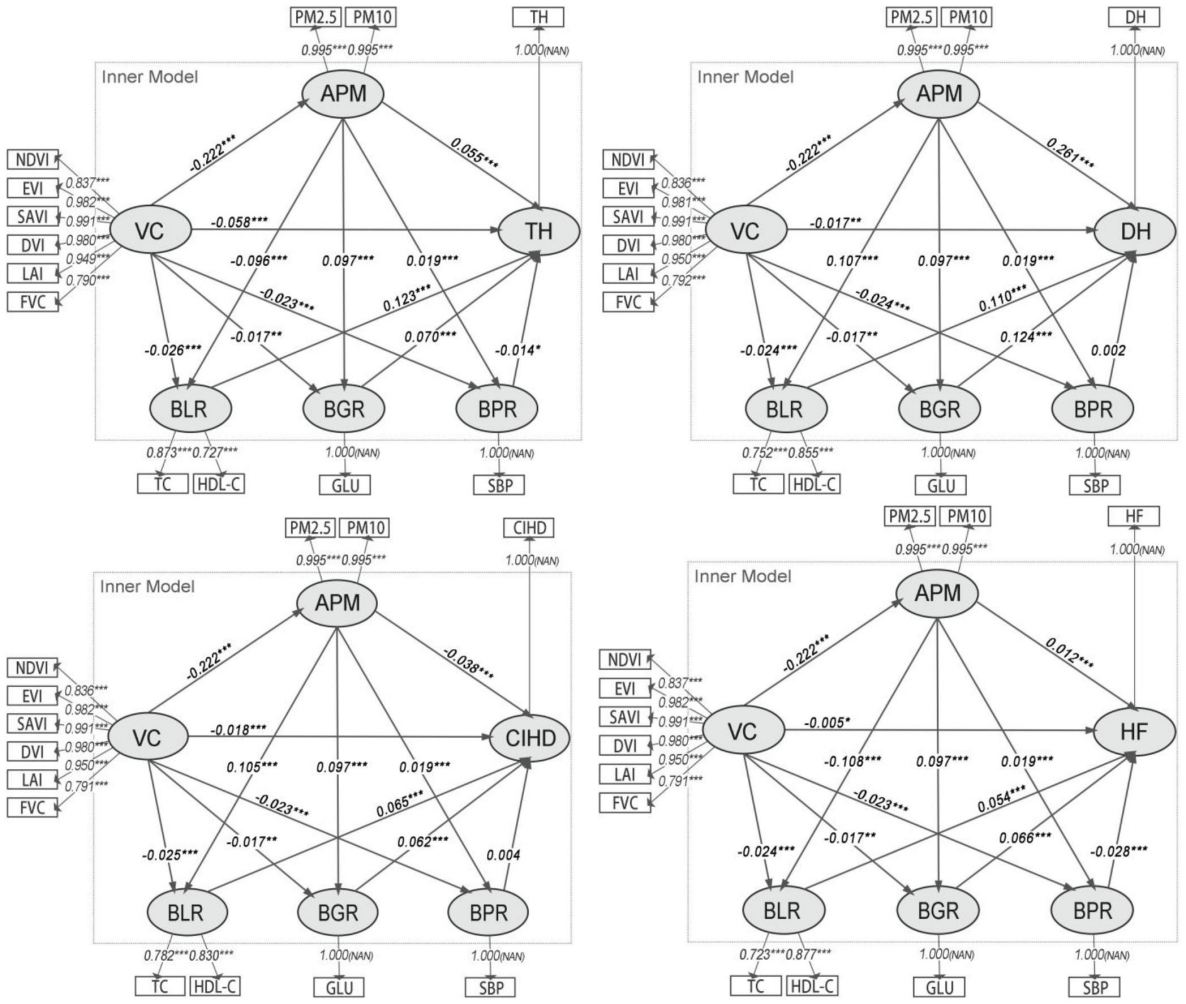
Path diagrams of the PLS-SEMs in model group 2. VC, Vegetation cover; APM, Physiological health risk; BLR, Blood lipid risk; BGR, Blood glucose risk; BPR, Blood pressure risk; IR, Inflammatory risk; TH, Times of hospitalizations; DH, Days of hospitalizations; CIHD, Diagnosed chronic ischemic heart disease; HF, Diagnosed heart failure.

The path results (**Figure 3**) confirm a significant negative association between VC and APM (β ≈ -0.222, p< 0.001). APM was significantly and positively associated with both BLR (β ≈ 0.115, p< 0.001) and BGR (β ≈ 0.100, p< 0.001), but its association with BPR was not significant. In turn, BLR and BGR were significantly associated with all four CHO variables (TH, DH, CIHD, HF). BPR was only significantly associated with HF (β = -0.005, p< 0.01).

The analysis of indirect associations (**Table 3**) indicates that the most substantial pathways from VC to CHOs operate through APM to BLR and BGR. For hospitalization outcomes, the indirect pathways involving BLR and BGR were strongest, with BGR being slightly more prominent for DH (Specific Indirect Effect = -0.00266) than BLR (Specific Indirect Effect = -0.00260). For disease diagnoses, the pathways via BLR and BGR were also the most prominent, with BLR being stronger for CIHD (Specific Indirect Effect = -0.00150) and BGR being stronger for HF (Specific Indirect Effect = -0.00141). The role of BPR as an intermediate variable was negligible in most models, showing only a minor significant indirect association for HF.

**Table 3:** Chain mediation effects of the PLS-SEMs in model group 2.

Model variable (PHRi)	Total effect	Direct effect	Specific indirect effect
Model 2a (VC→APM→PHR→TH)			
BLR	-0.07881^***^	-0.05845^***^	-0.00262^***^
BGR	-0.07881^***^	-0.05845^***^	-0.00150^***^
BPR	-0.07881^***^	-0.05845^***^	0.00005
Model 2b (VC→APM→PHR→DH)			
BLR	-0.08455^***^	-0.01651^**^	-0.00260^***^
BGR	-0.08455^***^	-0.01651^**^	-0.00266^***^
BPR	-0.08455^***^	-0.01651^**^	-0.00001
Model 2c (VC→APM→PHR→CIHD)			
BLR	-0.01536^***^	-0.01825^***^	-0.00150^***^
BGR	-0.01536^***^	-0.01825^***^	-0.00134^***^
BPR	-0.01536^***^	-0.01825^***^	-0.00002
Model 2d (VC→APM→PHR→HF)			
BLR	-0.01244^***^	-0.00544^*^	-0.00130^***^
BGR	-0.01244^***^	-0.00544^*^	-0.00141^***^
BPR	-0.01244^***^	-0.00544^*^	-0.00011^**^

VC, Vegetation cover; APM, Physiological health risk; PHR, Airborne particulate matter; BLR, Blood lipid risk; BGR, Blood glucose risk; BPR, Blood pressure risk; IR, Inflammatory risk; TH, Times of hospitalizations; DH, Days of hospitalizations; CIHD, Diagnosed chronic ischemic heart disease; HF, Diagnosed heart failure.

^*^p< 0.05; ^**^p< 0.01; ^***^p< 0.001. The underlined values in the table denote statistically significant and relatively large effect sizes and proportions, highlighting paths with substantial indirect effects. These paths and their corresponding variables are identified as having significant and strong mediating effects in the model, and are thus selected for further analysis or interpretation.

### 4.3 Associations involving disaggregated pm and biomarkers

Based on the findings from Model Group 2, the third stage of our analysis simultaneously disaggregated APM into PM2.5 and PM10, and the key PHR constructs into their constituent biomarkers (TC, HDL-C, and GLU). Model evaluation metrics remained robust (**Supplementary Table S3**).

**Figure 4:**
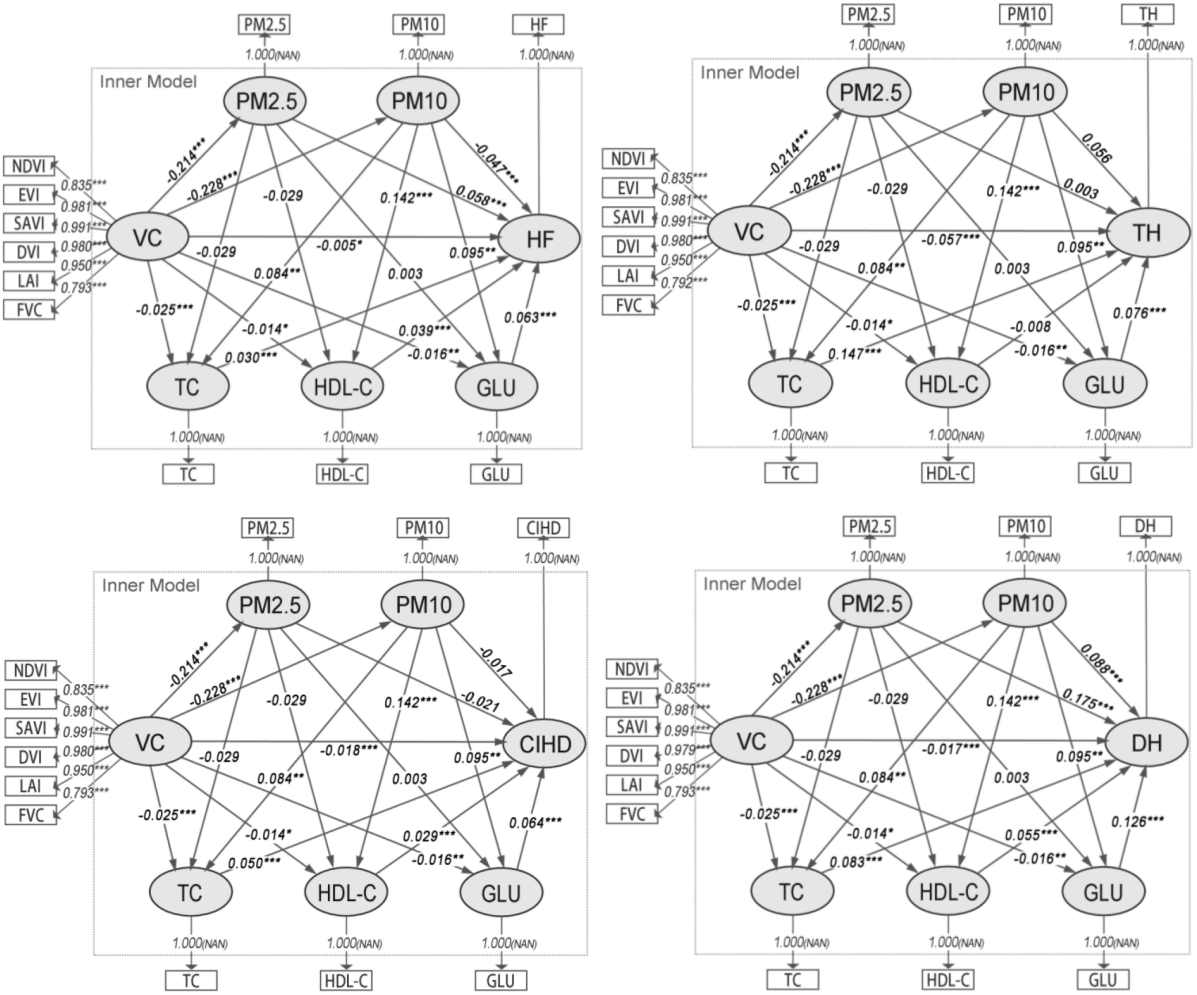
Path diagrams of the PLS-SEMs in model group 3. VC, Vegetation cover; TC, Total cholesterol; HDL-C, High density lipoprotein cholesterol; GLU, Blood glucose; TH, Times of hospitalizations; DH, Days of hospitalizations; CIHD, Diagnosed chronic ischemic heart disease; HF, Diagnosed heart failure.

The path analysis results (**Figure 4**) reveal a critical finding: while VC was significantly and negatively associated with both PM2.5 (β ≈ -0.214, p< 0.001) and PM10 (β ≈ -0.228, p< 0.001), only PM10 showed significant subsequent associations with the key biomarkers. Specifically, PM10 was positively associated with TC (β ≈ 0.064, p< 0.001) and GLU (β ≈ 0.100, p< 0.001), and negatively associated with HDL-C (β ≈ -0.119, p< 0.001). In contrast, the paths from PM2.5 to these biomarkers were not statistically significant.

Consequently, the analysis of indirect associations (**Table 4**) demonstrates that the significant sequential pathways from VC to all CHOs operate exclusively through PM10. For TH, the strongest indirect pathway involved PM10 and TC (Specific Indirect Effect = -0.00282, p< 0.01). For DH, CIHD, and HF, the pathways involving PM10 and GLU were consistently the most prominent (e.g., for DH: Specific Indirect Effect = -0.00273, p< 0.01). These results identify PM10 as the primary PM variable in this chain of association, linking VC to CHOs via specific metabolic biomarkers.

**Table 4:** Chain mediation effects of the PLS-SEMs in model group 3.

Model		Total effect	Direct effect	Specific indirect effect
Variable (APM)	Variable (PHR)			
MODEL 3a (VC→APM→BM→TH)				
PM2.5	TC	-0.07877^***^	-0.05717^***^	0.00092
	HDL-C	-0.07877^***^	-0.05717^***^	-0.00005
	GLU	-0.07877^***^	-0.05717^***^	-0.00004
PM10	TC	-0.07877^***^	-0.05717^***^	-0.00282^**^
	HDL-C	-0.07877^***^	-0.05717^***^	0.00026
	GLU	-0.07877^***^	-0.05717^***^	-0.00166^**^
MODEL 3b (VC→APM→BM→DH)				
PM2.5	TC	-0.08461^***^	-0.01690^***^	0.00052
	HDL-C	-0.08461^***^	-0.01690^***^	0.00034
	GLU	-0.08461^***^	-0.01690^***^	-0.00007
PM10	TC	-0.08461^***^	-0.01690^***^	-0.00159^**^
	HDL-C	-0.08461^***^	-0.01690^***^	-0.00176^***^
	GLU	-0.08461^***^	-0.01690^***^	-0.00273^**^
Model 3c (VC→APM→BM→CIHD)				
PM2.5	TC	-0.01532^***^	-0.01814^***^	0.00032
	HDL-C	-0.01532^***^	-0.01814^***^	0.00018
	GLU	-0.01532^***^	-0.01814^***^	-0.00004
PM10	TC	-0.01532^***^	-0.01814^***^	-0.00097^**^
	HDL-C	-0.01532^***^	-0.01814^***^	-0.00095^***^
	GLU	-0.01532^***^	-0.01814^***^	-0.00138^**^
Model 3d (VC→APM→BM→HF)				
PM2.5	TC	-0.01240^***^	-0.00546^*^	0.00019
	HDL-C	-0.01240^***^	-0.00546^*^	0.00025
	GLU	-0.01240^***^	-0.00546^*^	-0.00004
PM10	TC	-0.01240^***^	-0.00546^*^	-0.00058^**^
HDL-C	-0.01240^***^	-0.00546^*^	-0.00127^***^
GLU	-0.01240^***^	-0.00546^*^	-0.00137^**^

VC, Vegetation cover; APM, Physiological health risk; BM, Biomarker; TC, Total cholesterol; HDL-C, High density lipoprotein cholesterol; GLU, Blood glucose; TH, Times of hospitalizations; DH, Days of hospitalizations; CIHD, Diagnosed chronic ischemic heart disease; HF, Diagnosed heart failure.

^*^p< 0.05; ^**^p< 0.01; ^***^p< 0.001. The underlined values in the table denote statistically significant and relatively large effect sizes and proportions, highlighting paths with substantial indirect effects. These paths and their corresponding variables are identified as having significant and strong mediating effects in the model, and are thus selected for further analysis or interpretation.

### 4.4 Comparison of association strengths for individual VC indicators

In the final stage, we compared the relative strength of six individual VC indicators within the most salient pathway identified in Model Group 3. Based on those results, PM10 was used as the APM indicator, while TC was selected as the key biomarker for the TH outcome, and GLU was selected for the DH, CIHD, and HF outcomes.

The comprehensive-effect model was found to be invalid due to severe multicollinearity (VIF > 5), as anticipated in our pre-specified analytical plan. Consequently, we proceeded with single-factor models. The results (**Supplementary Figure S3** and **Table 5**) show that all six VC indicators were significantly and negatively associated with PM10. In the subsequent pathways to CHOs, LAI consistently emerged as the indicator with the strongest indirect association for disease diagnoses, particularly for CIHD (Specific Indirect Effect = -0.00164) and HF (Specific Indirect Effect = -0.00164). For hospitalization outcomes (TH and DH), SAVI and LAI showed the most prominent associations. Notably, FVC also demonstrated a strong association for TH (Specific Indirect Effect = -0.00172) and DH (Specific Indirect Effect = -0.00259), although slightly weaker than LAI. This suggests that vegetation quality and structure, as captured by LAI and FVC, are particularly important in this environmental health pathway.

**Table 5:** Chain mediation effects of the PLS-SEMs in model group 4.

Model		Total effect	Direct effect	Specific indirect effect
Variable (CHO)	Variable (VC)			
TH	NDVI	-0.05206^***^	-0.03957^***^	-0.00082^***^
	EVI	-0.08096^***^	-0.05993^***^	-0.00189^***^
	SAVI	-0.08576^***^	-0.06425^***^	-0.00193^***^
	DVI	-0.08897^***^	-0.06853^***^	-0.00180^***^
	LAI	-0.07046^***^	-0.04899^***^	-0.00193^***^
	FVC	-0.04723^***^	-0.03263^***^	-0.00172^***^
DH	NDVI	-0.05143^***^	-0.02109^***^	-0.00129^***^
	EVI	-0.08527^***^	-0.01655^*^	-0.00311^***^
	SAVI	-0.08864^***^	-0.01772^***^	-0.00320^***^
	DVI	-0.08600^***^	-0.01960^***^	-0.00298^***^
	LAI	-0.08155^***^	-0.01321^*^	-0.00321^***^
	FVC	-0.06547^***^	-0.01245^*^	-0.00259^***^
CIHD	NDVI	-0.01181^***^	-0.01227^***^	-0.00066^***^
	EVI	-0.01737^***^	-0.02213^***^	-0.00159^***^
	SAVI	-0.01778^***^	-0.02276^***^	-0.00163^***^
	DVI	-0.01884^***^	-0.02323^***^	-0.00152^***^
	LAI	-0.01636^***^	-0.02177^***^	-0.00164^***^
	FVC	0.00107	-0.00283	-0.00132^***^
HF	NDVI	-0.01688^***^	-0.01309^***^	-0.00066^***^
EVI	-0.01306^***^	-0.00651^**^	-0.00159^***^
SAVI	-0.01341^***^	-0.00666^**^	-0.00164^***^
DVI	-0.01357^***^	-0.00709^**^	-0.00153^***^
LAI	-0.00938^***^	-0.00320	-0.00164^***^
FVC	-0.00390	0.00063	-0.00132^***^

CHO, Cardiovascular health outcome; VC, Vegetation cover; TH, Times of hospitalizations; DH, Days of hospitalizations; CIHD, Diagnosed chronic ischemic heart disease; HF, Diagnosed heart failure; NDVI, Normalized difference vegetation index; EVI, Enhanced vegetation index; SAVI, Soil adjusted vegetation index; DVI, Difference vegetation index; FVC, Fractional Vegetation Cover; LAI, Leaf area index.

^*^p< 0.05; ^**^p< 0.01; ^***^p< 0.001. The underlined values in the table denote statistically significant and relatively large effect sizes and proportions, highlighting paths with substantial indirect effects. These paths and their corresponding variables are identified as having significant and strong mediating effects in the model, and are thus selected for further analysis or interpretation.

## 5 Discussion

### 5.1 Interpretation of key associative pathways

The results from our four-stage analytical strategy consistently support an association between VC and CHOs, which is statistically explained by indirect pathways involving APM and PHR. This highlights the complex web of associations through which green spaces are linked to cardiovascular health.

A central finding, established in Model Group 3, is the dominant role of PM10. While VC was associated with reductions in both PM2.5 and PM10 concentrations, only the pathways involving PM10 were significantly linked to subsequent changes in metabolic biomarkers and CHOs. This differential impact could be attributed to PM10 being more easily captured by vegetation. Additionally, PM10’s deposition in the upper respiratory tract may more readily trigger systemic responses affecting lipid and glucose metabolism.

The analysis further specified the biological pathways involved. Model Group 3 demonstrated that lower PM10 concentrations were associated with reduced TC and GLU levels, and increased HDL-C levels. These biomarkers, in turn, were linked to better health outcomes. Notably, the indirect pathways showed specificity: the VC→PM10→TC pathway was most prominent for TH, whereas the VC→PM10→GLU pathway was strongest for DH, CIHD, and HF. This suggests that PM10 exposure may influence hospitalization frequency and disease severity through distinct metabolic routes. The significant role of HDL-C in the pathways for DH, CIHD, and HF, but not for TH, further underscores this complexity.

Finally, the single-factor models in Model Group 4 confirmed that while all vegetation indicators were associated with reduced PM10, LAI and FVC showed the most significant indirect associations with CHOs. This indicates that vegetation structure and density are key characteristics. LAI, reflecting the total leaf surface area, is directly related to the capacity for air purification and particle deposition. FVC represents the density of VC, which also contributes to overall air quality improvement.

These findings collectively underscore the multifaceted nature of urban greening’s health benefits. They suggest that urban planning strategies should prioritize vegetation types that effectively reduce PM10 concentrations. Based on this study’s results, increasing LAI and FVC could be effective strategies for improving cardiovascular health. It is also important to note the presence of inconsistent associations in some pathways, such as the VC→APM→CVDD path in Model 1b. This phenomenon, potentially a suppressor effect, suggests the existence of complex underlying relationships not fully captured by the current model and highlights a valuable direction for future research.

### 5.2 Comparison with existing literature

Our general finding that green space is associated with better cardiovascular health via pathways involving PM and metabolic biomarkers is consistent with a growing body of literature. For example, studies have similarly identified PM as a key intermediate variable in the link between greenness and atherosclerotic CVD risk (Shen and Lung, 2016; Dong et al., 2021), and have associated green space with improved lipid profiles (R. Lei et al., 2024; Xu et al., 2022, better glucose metabolism (Yang et al., 2019; Li et al., 2021), and reduced inflammation (Egorov et al., 2024; Lai et al., 2024). Our study builds upon this foundation by using a path analysis framework to explore these sequential relationships, aligning with findings that cardiometabolic disorders mediate the relationship between green space and CVD (Yang et al., 2020).

A notable finding of our study, confirmed robustly in our competitive model (Model Group 3), is the dominant role of PM10 over PM2.5 in the associative pathways. This diverges from several studies that have specifically implicated PM2.5 as the primary mediator (Li et al., 2022; Lei et al., 2024). Several factors could explain this discrepancy. First, urban vegetation may be more effective at capturing larger PM10 particles through deposition and impaction (Diener and Mudu, 2021). Second, our patient cohort might be particularly susceptible to the inflammatory and metabolic effects induced by PM10. While PM2.5 is known for its ability to penetrate deeper into the bloodstream, the metabolic responses observed in our cross-sectional data may be more strongly linked to PM10 exposure.

Furthermore, our granular analysis pinpointed TC and GLU as the most significant biomarkers. While previous research has broadly linked greenness to cardiometabolic disorders (Yang et al., 2020), our study identifies TC and GLU as particularly sensitive indicators in this environmental health context. The strong association with GLU aligns with studies suggesting that air pollution can induce insulin resistance and impair glucose metabolism (Yang et al., 2018; Burkart et al., 2022). The specific prominence of TC in the pathway to TH, and the significant roles of both TC and HDL-C in other outcomes, suggests that PM10 exposure in this population may have a pronounced effect on overall cholesterol regulation, a finding that complements broader evidence linking air pollution to adverse lipid profiles (Cesaroni et al., 2014). This specificity provides a more focused target for future mechanistic research, suggesting that TC and GLU could serve as key biomarkers for assessing the cardiovascular benefits of urban greening initiatives.

### 5.3 Methodological contributions and reflections

This study presented several methodological innovations for exploring the complex relationships between green space, air quality, PHR, and CHOs. (1) While traditional structural equation modeling has been widely used in previous epidemiological studies (Hays et al., 2005; Grande et al., 2020), PLS-SEMs have gained popularity in recent years for their efficiency at handling complex structural models with multiple constructs, indicators, and model relationships. For instance, some research employed PLS-SEMs to investigate the relationships between green structures, air pollution, temperature, and CVD mortality (Shen and Lung, 2016). Our study builds upon this trend, leveraging the ability of PLS-SEMs to explore more complex mediation pathways. (2) A key innovation in our approach is the application of chain mediation analysis to examine the mechanisms linking green space to cardiovascular health. While some previous studies have considered potential mediators between environmental factors and health outcomes, they typically focused on single mediators such as air pollution or physical activity (Bauwelinck et al., 2021; Yu et al., 2023). Our study extends this by incorporating multiple potential mediators—air quality and PHR—in a chain mediation model. This approach allows for a more comprehensive understanding of the complex pathways through which green space influences cardiovascular health. (3) Our study employed a series of increasingly refined models (Model Groups 1–4) to elucidate the associations at play: Model Group 1 established the overall associative pathway, Model Group 2 deconstructed the composite PHR into its sub-risks, Model Group 3 simultaneously examined the roles of different PM sizes (PM2.5 and PM10) and specific biomarkers (TC, HDL-C, GLU) in a competitive model, and Model Group 4 explored the relative importance of various individual vegetation indicators. This progressive refinement allowed for a more nuanced and comprehensive analysis of the relationships between variables. (4) In Model Groups 2–4, we considered both the interactive effects of multiple variables in chain mediation models and the individual effects of single indicators. This dual approach, using both comprehensive-effect and single-factor models, provides a more realistic representation of complex real-world mechanisms while also allowing for the identification of key individual factors. Our study employed different models based on the specific requirements of each analysis stage. This flexible approach, using comprehensive-effect models as the primary strategy with single-factor models as a pre-specified alternative to address multicollinearity, was applied in Model Groups 2, 3, and 4. This flexible approach to model selection enhanced the scientific rigor and accuracy of our findings. In conclusion, our methodological approach—combining PLS-SEMs with chain mediation analysis and a progressive model refinement strategy—offers a novel and comprehensive framework for investigating the complex relationships between environmental factors and health outcomes. This approach allows for a more detailed and nuanced understanding of the mechanisms linking green space to cardiovascular health, and it provides valuable insights for urban planning and public health strategies.

### 5.4 Limitations

This study has several important limitations that must be considered when interpreting the findings. First and foremost, the cross-sectional and retrospective nature of our study design is a major limitation. The environmental exposures (VC and PM) were assigned based on annual averages for the year preceding hospitalization, and physiological data were collected during the hospitalization episode. This design does not allow for the establishment of clear temporal precedence, which is a prerequisite for making causal claims. Consequently, our findings should be interpreted as evidence of statistical association rather than causal mediation. The language used throughout this paper, such as indirect association and pathway of association, reflects this non-causal, exploratory framework. Second, our analysis did not include several crucial individual-level confounders. Data on patient age, sex, socioeconomic status, lifestyle factors such as smoking and diet, and pre-existing comorbidities were not available in the electronic medical records used for this study. These factors are known to be strong predictors of both residential location choices and cardiovascular risk. Their omission means that the associations we observed could be biased due to unmeasured confounding. For example, higher socioeconomic status might be associated with both living in greener neighborhoods and better baseline health, which could partially explain the observed link between vegetation and better health outcomes. Therefore, the results should be interpreted with caution. Third, the use of annual average data for environmental exposures, while common in such studies, may mask the potentially important effects of short-term or seasonal fluctuations in PM concentrations. The health impacts of acute exposure spikes might differ from those of chronic, long-term exposure. Finally, while we used a 1000m buffer to estimate exposure, this is an approximation and may not perfectly reflect an individual’s true exposure, which is influenced by daily mobility patterns, time spent indoors versus outdoors, and occupational exposures. Future research employing longitudinal designs, with detailed individual-level data on confounders and time-activity patterns, is needed to confirm the exploratory findings of this study and to establish causal relationships.

## 6 Conclusions

This study elucidates a key environmental health pathway, demonstrating a significant association between residential VC and cardiovascular health that is statistically explained by a sequential path involving air quality and metabolic biomarkers. Our analysis robustly identifies PM10, over the more commonly implicated PM2.5, as the primary atmospheric intermediary linking vegetation to physiological risk. Furthermore, our findings decompose this association into distinct metabolic routes. We demonstrate that the pathway from PM10 to cardiovascular outcomes is statistically specified through key biomarkers, with TC being most prominent for TH, while GLU is the crucial link to DH and specific diagnoses like CIHD and HF. Crucially, these health-promoting pathways are anchored in the quality and structure of urban vegetation. LAI and FVC emerged as the most significant vegetation characteristics, suggesting that dense, structurally complex green spaces are most effective at initiating this beneficial cascade. These findings have direct implications for urban planning and public health, indicating that strategic greening policies focused on maximizing LAI and FVC can be a potent tool for targeted air quality management and cardiovascular disease prevention. Future longitudinal research is essential to validate these associative pathways and explore their causal underpinnings.

## Data Availability

The original contributions presented in the study are included in the article/**Supplementary Material**. Further inquiries can be directed to the corresponding authors.
