# Supplementary material for: Associations among vegetation cover, particulate matter, and cardiovascular health in urban environments: a path analysis
Source: Front Plant Sci. 2025 Nov 7;16:1659005. doi: 10.3389/fpls.2025.1659005 (PMC12634656; doi:10.3389/fpls.2025.1659005)
Supplement: Supplementary file 1 [file DataSheet1.pdf]

## Appendix A

### 1. Vegetation cover (VC)

For an accurate and comprehensive assessment of the vegetation cover (VC) around patients' residences, this study employed six vegetation indices as observed variables: the normalized difference vegetation index (NDVI), the enhanced vegetation index (EVI), the soil adjusted vegetation index (SAVI), the difference vegetation index (DVI), fractional vegetation cover (FVC), and the leaf area index (LAI). The NDVI has been the most widely used indicator in previous studies on the relationship between green space levels and health outcomes (Gullon et al., 2023, Wang et al., 2019). EVI (Yu et al., 2023, Baheti et al., 2023) and SAVI (Luo et al., 2022) have been utilized in some studies to reflect vegetation growth levels. Although the DVI, LAI, and FVC are less commonly applied in epidemiological research, each has unique characteristics: the DVI effectively reflects changes in VC, the LAI is a crucial parameter for expressing the eco-physiological processes and growth of vegetation and is useful for calculating vegetation's air pollution removal effects (Fusaro et al., 2017, Manes et al., 2016), and FVC is widely used to describe vegetation quality and ecosystem changes in climate, hydrological, and geochemical cycles (Jiménez-Muñoz et al., 2009, Song et al., 2017).

For image data acquisition and processing, we used atmospherically corrected surface reflectance data from the Landsat 8 OLI/TIRS sensor with a 30 m spatial resolution. Using the Google Earth Engine platform, satellite images with less than 20% annual cloud cover were selected, and relevant algorithms from previous studies (Table 1) were applied to calculate annual averages of the above vegetation indices from 2013 to 2022. To capture more detailed green space changes, the data were resampled to a 10 m resolution. Subsequently, ENVI software (Version 5.6, NV5 Geospatial Solutions, USA) was used for preprocessing, including the removal of outliers, setting water body missing values, and normalization (Markevych et al., 2016). Considering the dynamic nature of VC and referencing previous studies (Zhou et al., 2023, Li et al., 2022), we selected a one-year timeframe for VC statistics. Additionally, taking into account the impact of VC on air quality (Venter et al., 2024), potential patient activity range, and air

quality indicator resolution, a 1000 m spatial range was chosen for statistical analysis. Using ArcGIS Pro software (Version 3.2, Esri, USA), we calculated the annual average values of each vegetation index within a 1000 m buffer zone around patients' addresses for the calendar year preceding their hospital admission.

## 2. Air particulate matter (APM)

Given the established impact of VC on air quality, particularly particulate matter (Diener and Mudu, 2021), we selected PM2.5 and PM10 as observational variables for air particulate matter (APM). Data were sourced from the China High Air Pollutants (CHAP) dataset. This dataset employs artificial intelligence techniques to fill spatial gaps in MODIS MAIAC AOD products, integrating ground-based observations, atmospheric reanalysis, and emission inventories to generate seamless ground-level PM2.5 and PM10 data for China. Ten-fold cross-validation of the data demonstrated determination coefficients ($R^2$) of 0.92 and 0.90 and root mean square errors (RMSEs) of 10.76 μg/m3 and 21.12 $\mu g/m^3$ for PM2.5 and PM10, respectively (Wei et al., 2021b, Wei et al., 2021a). Using ArcGIS Pro software, we resampled the spatial resolution from 1 km to 100 m and calculated the annual average concentrations of these two particulate matters within a 1000 m buffer zone around patients' addresses for the calendar year preceding their hospital admission, considering the range of VC's influence on air quality.

## 3. Physiological health risk (PHR)

Based on potential physiological health risks (PHRs) leading to adverse cardiovascular outcomes (Hu, 2023), we selected lipid risk (BLR), glucose risk (BGR), blood pressure risk (BPR), and inflammation risk (IR) as second-level latent variables for PHR, with corresponding biomarkers as observed variables (Table 1). For lipid risk, total cholesterol (TC) and high-density lipoprotein cholesterol (HDL-C) were chosen. As both elevated TC and reduced HDL-C are CVD risk factors (Zhu et al., 2016), and HDL-C levels are negatively correlated with atherosclerotic CVD risk (Gotto and Brinton, 2004), the HDL-C data were negatively transformed to ensure that an increase in any indicator represented increased risk. Blood glucose (GLU) was selected as the glucose risk indicator, effectively

complementing triglyceride and LDL level information (Poznyak et al., 2022). Systolic (SBP) and diastolic blood pressure (DBP) were chosen as blood pressure risk variables. For inflammation risk, lactate dehydrogenase (LDH) and C-reactive protein (CRP) were selected. LDH has predictive value for inflammatory disease severity (Gupta, 2022), while CRP is a potential risk factor for cardiovascular and cerebrovascular diseases in Chinese populations (Ye et al., 2007) and is commonly used as a clinical marker of inflammation (Ridker et al., 2002).

## 4. Cardiovascular health outcomes (CHOs)

We selected hospitalization utilization (HU) and CVD diagnosis (CVDD) as latent variables for cardiovascular health outcomes (CHOs). Hospitalization utilization was reflected by the number of hospitalization days (DH) and times (TH) reported in patients' electronic medical records, used to assess disease severity and recovery control. CVDD was based on diagnoses reported in patients' electronic medical records, referencing the International Classification of Diseases, 10th Revision, Clinical Modification (ICD-10-CM). Chronic ischemic heart disease (CIHD, code I25) and heart failure (HF, code I50) from the circulatory system disease category were chosen as observational variables. These two diseases were selected because they represent significant types of CVDs and can reflect long-term cardiovascular health status.

## References


BAHETI, B., CHEN, G. B., DING, Z. A., WU, R. Y., ZHANG, C. Y., ZHOU, L., LIU, X. T., SONG, X. Q. & WANG, C. J. 2023. Residential greenness alleviated the adverse associations of long-term exposure to ambient PM with cardiac conduction abnormalities in rural adults. *Environmental Research,* 237.

DIENER, A. & MUDU, P. 2021. How can vegetation protect us from air pollution? A critical review on green spaces' mitigation abilities for air-borne particles from a public health perspective-with implications for urban planning. *Science of the Total Environment,* 796.

FUSARO, L., MARANDO, F., SEBASTIANI, A., CAPOTORTI, G., BLASI, C., COPIZ, R., CONGEDO, L., MUNAFò, M., CIANCARELLA, L. & MANES, F. 2017. Mapping and Assessment of PM<sub>10</sub> and O<sub>3</sub> Removal by Woody Vegetation at Urban and Regional Level. *Remote Sensing,* 9.

GOTTO, A. M. & BRINTON, E. A. 2004. Assessing low levels of high-density lipoprotein cholesterol

as a risk factor in coronary heart disease - A working group report and update. *Journal of the American College of Cardiology,* 43, 717-724.

GULLON, P., FONTAN-VELA, M., DIEZ, J., NIEUWENHUIJSEN, M., ROJAS-RUEDA, D., ESCOBAR, F. & FRANCO, M. 2023. Who benefits from green spaces? Surrounding greenness and incidence of cardiovascular disease in a population-based electronic medical records cohort in Madrid. *International Journal of Hygiene and Environmental Health,* 252.

GUPTA, G. S. 2022. The Lactate and the Lactate Dehydrogenase in Inflammatory Diseases and Major Risk Factors in COVID-19 Patients. *Inflammation,* 45, 2091-2123.

HU, S. S. 2023. The Writing Committee of the Report on Cardiovascular Health Diseases in China. 28, 297-312.

JIMéNEZ-MUñOZ, J. C., SOBRINO, J. A., PLAZA, A., GUANTER, L., MORENO, J. & MARTíNEZ, P. 2009. Comparison Between Fractional Vegetation Cover Retrievals from Vegetation Indices and Spectral Mixture Analysis: Case Study of PROBA/CHRIS Data Over an Agricultural Area. *Sensors,* 9, 768-793.

LI, T. Z., YU, Z. B., XU, L. S., WU, Y. H., YU, L. H., YANG, Z. M., SHEN, P., LIN, H. B., SHUI, L. M., TANG, M. L., JIN, M. J., CHEN, K. & WANG, J. B. 2022. Residential greenness, air pollution, and incident ischemic heart disease: A prospective cohort study in China. *Science of the Total Environment,* 838.

LUO, Y. N., YANG, B. Y., ZOU, Z. Y., MARKEVYCH, I., BROWNING, M. H. E. M., HEINRICH, J., BAO, W. W., GUO, Y. M., HU, L. W., CHEN, G. B., MA, J., MA, Y. H., CHEN, Y. J. & DONG, G. H. 2022. Associations of greenness surrounding schools with blood pressure and hypertension: A nationwide cross-sectional study of 61,229 children and adolescents in China. *Environmental Research,* 204.

MANES, F., MARANDO, F., CAPOTORTI, G., BLASI, C., SALVATORI, E., FUSARO, L., CIANCARELLA, L., MIRCEA, M., MARCHETTI, M., CHIRICI, G. & MUNAFO, M. 2016. Regulating Ecosystem Services of forests in ten Italian Metropolitan Cities: Air quality improvement by PM<sub>10</sub> and O<sub>3</sub> removal. *Ecological Indicators,* 67, 425-440.

MARKEVYCH, I., STANDL, M., SUGIRI, D., HARRIS, C., MAIER, W., BERDEL, D. & HEINRICH, J. 2016. Residential greenness and blood lipids in children: A longitudinal analysis in GINIplus and LISAplus. *Environmental Research,* 151, 168-173.

POZNYAK, A. V., LITVINOVA, L., POGGIO, P., SUKHORUKOV, V. N. & OREKHOV, A. N. 2022. Effect of Glucose Levels on Cardiovascular Risk. *Cells,* 11.

RIDKER, P. M., RIFAI, N., ROSE, L., BURING, J. E. & COOK, N. R. 2002. Comparison of C-reactive protein and low-density lipoprotein cholesterol levels in the prediction of first cardiovascular events. *New England Journal of Medicine,* 347, 1557-1565.

SONG, W., MU, X., RUAN, G., GAO, Z., LI, L. & YAN, G. 2017. Estimating fractional vegetation cover and the vegetation index of bare soil and highly dense vegetation with a physically based method. *International Journal of Applied Earth Observation and Geoinformation,* 58, 168-176.

VENTER, Z. S., HASSANI, A., STANGE, E., SCHNEIDER, P. & CASTELL, N. 2024. Reassessing the

role of urban green space in air pollution control. *Proceedings of the National Academy of Sciences of the United States of America,* 121.

WANG, K. F., LOMBARD, J., RUNDEK, T., DONG, C. H., GUTIERREZ, C. M., BYRNE, M. M., TORO, M., NARDI, M. I., KARDYS, J., YI, L., SZAPOCZNIK, J. & BROWN, S. C. 2019. Relationship of Neighborhood Greenness to Heart Disease in 249 405 US Medicare Beneficiaries. *Journal of the American Heart Association,* 8.

WEI, J., LI, Z. Q., LYAPUSTIN, A., SUN, L., PENG, Y. R., XUE, W. H., SU, T. N. & CRIBB, M. 2021a. Reconstructing 1-km-resolution high-quality PM<sub>2.5</sub> data records from 2000 to 2018 in China: spatiotemporal variations and policy implications. *Remote Sensing of Environment,* 252.

WEI, J., LI, Z. Q., XUE, W. H., SUN, L., FAN, T. Y., LIU, L., SU, T. N. & CRIBB, M. 2021b. The ChinaHighPM<sub>10</sub> dataset: generation, validation, and spatiotemporal variations from 2015 to 2019 across China. *Environment International,* 146.

YE, X. W., YU, Z. J., LI, H. X., FRANCO, O. H., LIU, Y. & LIN, X. 2007. Distributions of C-reactive protein and its association with metabolic syndrome in middle-aged and older Chinese people. *Journal of the American College of Cardiology,* 49**,** 1798-1805.

YU, W., LIU, Z., LA, Y., FENG, C., YU, B., WANG, Q., LIU, M., LI, Z., FENG, Y., CIREN, L., ZENG, Q., ZHOU, J., ZHAO, X., JIA, P. & YANG, S. 2023. Associations between residential greenness and the predicted 10-year risk for atherosclerosis cardiovascular disease among Chinese adults. *Science of The Total Environment,* 868.

ZHOU, W. S., WANG, Q., LI, R., KADIER, A., WANG, W. J., ZHOU, F. F. & LING, L. 2023. Combined effects of heatwaves and air pollution, green space and blue space on the incidence of hypertension: A national cohort study. *Science of the Total Environment,* 867.

ZHU, J., GAO, R., ZHAO, S., LU, G., ZHAO, D. & LI, J. 2016. Guidelines for prevention and treatment of dyslipidemia in Chinese adults (revised 2016). *China Circulation Journal,* 31**,** 937-953.

**Appendix B**

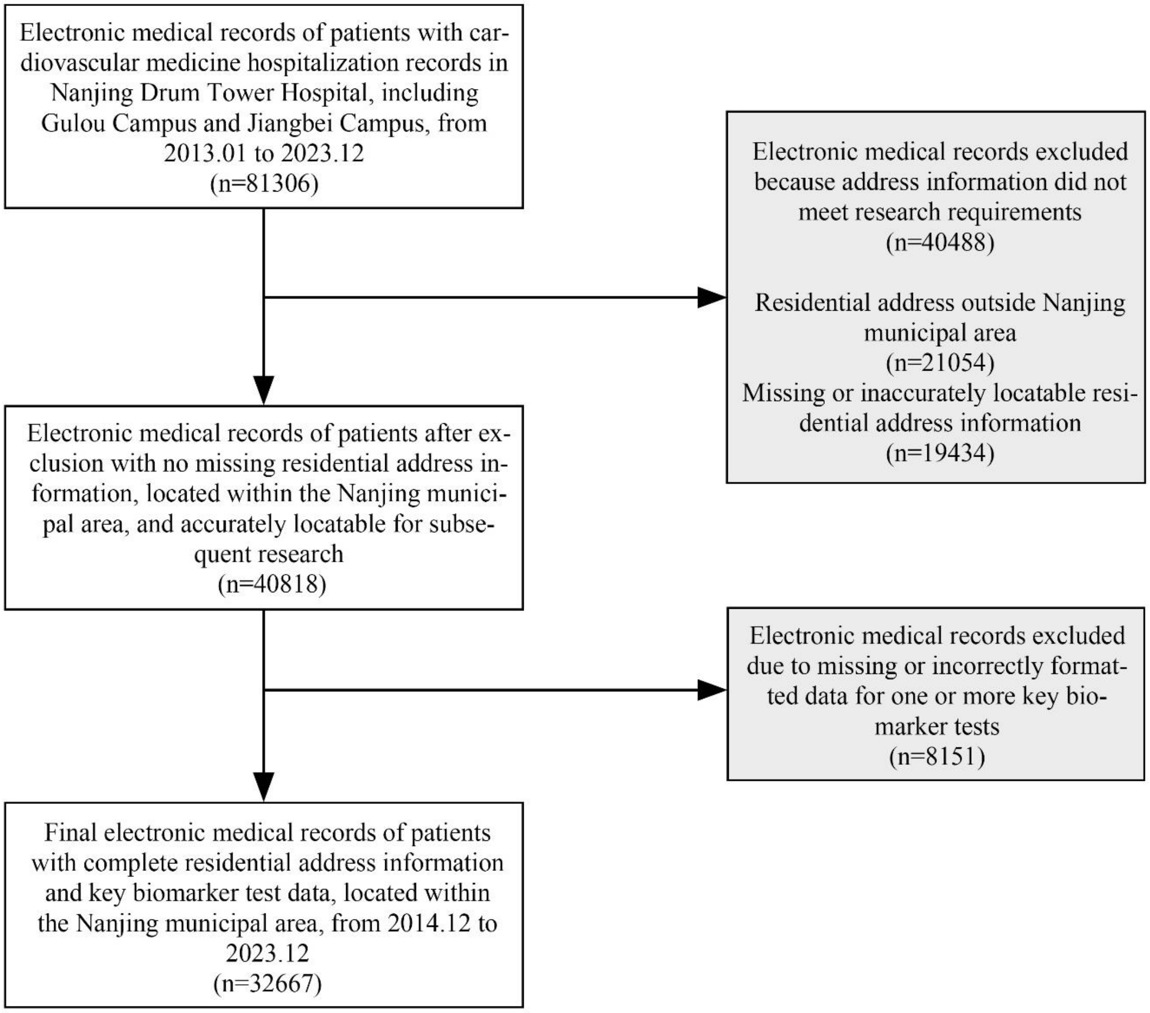


**Figure S1.** The participant selection process.

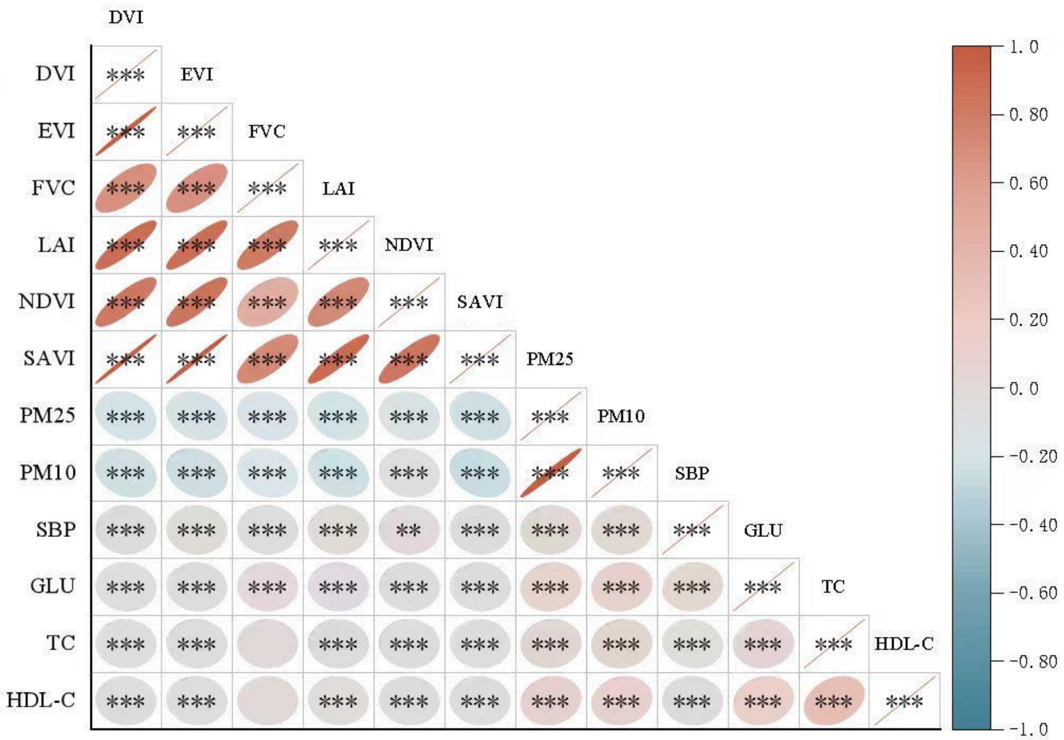


Note, *p≤0.05, **p≤0.01, ***p≤0.001

**Figure S2.** The bivariate correlations between all observed variables.

**Table S1** Performance evaluation of the PLS-SEMs in Model group 1.

| Model Variable | CR | AVE | Cronbach's α | (adj.) $R^2$ |
|---|---|---|---|---|
| **Model 1a (VC→APM→PHR→HU)** | | | | |
| VC | 0.972 | 0.856 | 0.965 | - |
| APM | 0.995 | 0.990 | 0.990 | 0.049 |
| PHR | 0.605 | 0.332 | 0.245 | 0.020 |
| HU | 0.721 | 0.578 | 0.315 | 0.120 |
| **Model 1b (VC→APM→PHR→CVDD)** | | | | |
| VC | 0.972 | 0.856 | 0.965 | - |
| APM | 0.995 | 0.990 | 0.990 | 0.049 |
| PHR | 0.578 | 0.332 | 0.245 | 0.020 |
| CVDD | 0.704 | 0.543 | 0.158 | 0.080 |

Abbreviation, VC (Vegetation cover), APM (Physiological health risk), PHR (Airborne particulate matter), HU (Hospital utilization), CVDD (Cardiovascular disease diagnosis).

**Table S2** Performance evaluation of the PLS-SEMs in Model group 2.

| Model Variable | CR | AVE | Cronbach's α | (adj.) $R^2$ |
|---|---|---|---|---|
| **MODEL 2a (VC→APM→PHRi→TH)** | | | | |
| VC | 0.972 | 0.856 | 0.965 | - |
| APM | 0.995 | 0.990 | 0.990 | 0.049 |
| BLR | 0.783 | 0.646 | 0.463 | 0.011 |
| BGR | 1.000 | 1.000 | 1.000 | 0.010 |
| BPR | 1.000 | 1.000 | 1.000 | 0.001 |
| TH | 1.000 | 1.000 | 1.000 | 0.033 |
| **MODEL 2b (VC→APM→PHRi→DH)** | | | | |
| VC | 0.972 | 0.856 | 0.965 | - |
| APM | 0.995 | 0.990 | 0.990 | 0.049 |
| BLR | 0.786 | 0.648 | 0.463 | 0.013 |
| BGR | 1.000 | 1.000 | 1.000 | 0.010 |
| BPR | 1.000 | 1.000 | 1.000 | 0.001 |
| DH | 1.000 | 1.000 | 1.000 | 0.115 |
| **MODEL 2c (VC→APM→PHRi→CIHD)** | | | | |
| VC | 0.972 | 0.856 | 0.965 | - |
| APM | 0.995 | 0.990 | 0.990 | 0.049 |
| BLR | 0.788 | 0.650 | 0.463 | 0.013 |
| BGR | 1.000 | 1.000 | 1.000 | 0.010 |
| BPR | 1.000 | 1.000 | 1.000 | 0.001 |
| CIHD | 1.000 | 1.000 | 1.000 | 0.041 |
| **MODEL 2d (VC→APM→PHRi→HF)** | | | | |
| VC | 0.972 | 0.856 | 0.965 | - |
| APM | 0.995 | 0.990 | 0.990 | 0.049 |
| BLR | 0.783 | 0.645 | 0.463 | 0.014 |
| BGR | 1.000 | 1.000 | 1.000 | 0.010 |
| BPR | 1.000 | 1.000 | 1.000 | 0.001 |
| HF | 1.000 | 1.000 | 1.000 | 0.058 |

Abbreviation, VC (Vegetation cover), APM (Physiological health risk), PHR (Airborne particulate matter), BLR (Blood lipid risk), BGR (Blood glucose risk), BPR (Blood pressure risk), IR (Inflammatory risk), TH (Times of hospitalizations), DH (Days of hospitalizations), CIHD (Diagnosed chronic ischemic heart disease), HF (Diagnosed heart failure).

**Table S3** Performance evaluation of the PLS-SEMs in Model group 3.

| Model Variable | CR | AVE | Cronbach's α | (adj.) $R^2$ |
|---|---|---|---|---|
| **Model 3a (VC→APM→PHR→TH)** | | | | |
| VC | 0.972 | 0.855 | 0.965 | - |
| PM2.5 | 1.000 | 1.000 | 1.000 | 0.046 |
| PM10 | 1.000 | 1.000 | 1.000 | 0.052 |
| TC | 1.000 | 1.000 | 1.000 | 0.004 |
| HDL-C | 1.000 | 1.000 | 1.000 | 0.014 |
| GLU | 1.000 | 1.000 | 1.000 | 0.010 |
| TH | 1.000 | 1.000 | 1.000 | 0.039 |
| **Model 3b (VC→APM→PHR→DH)** | | | | |
| VC | 0.972 | 0.855 | 0.965 | - |
| PM2.5 | 1.000 | 1.000 | 1.000 | 0.046 |
| PM10 | 1.000 | 1.000 | 1.000 | 0.052 |
| TC | 1.000 | 1.000 | 1.000 | 0.004 |
| HDL-C | 1.000 | 1.000 | 1.000 | 0.014 |
| GLU | 1.000 | 1.000 | 1.000 | 0.010 |
| DH | 1.000 | 1.000 | 1.000 | 0.115 |
| **Model 3c (VC→APM→PHR→CIHD)** | | | | |
| VC | 0.972 | 0.855 | 0.965 | - |
| PM2.5 | 1.000 | 1.000 | 1.000 | 0.046 |
| PM10 | 1.000 | 1.000 | 1.000 | 0.052 |
| TC | 1.000 | 1.000 | 1.000 | 0.004 |
| HDL-C | 1.000 | 1.000 | 1.000 | 0.014 |
| GLU | 1.000 | 1.000 | 1.000 | 0.010 |
| CIHD | 1.000 | 1.000 | 1.000 | 0.041 |
| **Model 3d (VC→APM→PHR→HF)** | | | | |
| VC | 0.972 | 0.855 | 0.965 | - |
| PM2.5 | 1.000 | 1.000 | 1.000 | 0.046 |
| PM10 | 1.000 | 1.000 | 1.000 | 0.052 |
| TC | 1.000 | 1.000 | 1.000 | 0.004 |
| HDL-C | 1.000 | 1.000 | 1.000 | 0.014 |
| GLU | 1.000 | 1.000 | 1.000 | 0.010 |
| HF | 1.000 | 1.000 | 1.000 | 0.054 |

Abbreviation, VC (Vegetation cover), APM (Physiological health risk), PHR (Airborne particulate matter), BLR (Blood lipid risk), BGR (Blood glucose risk), BPR (Blood pressure risk), IR (Inflammatory risk), TH (Times of hospitalizations), DH (Days of hospitalizations), CIHD (Diagnosed chronic ischemic heart disease), HF (Diagnosed heart failure).

**(a)**

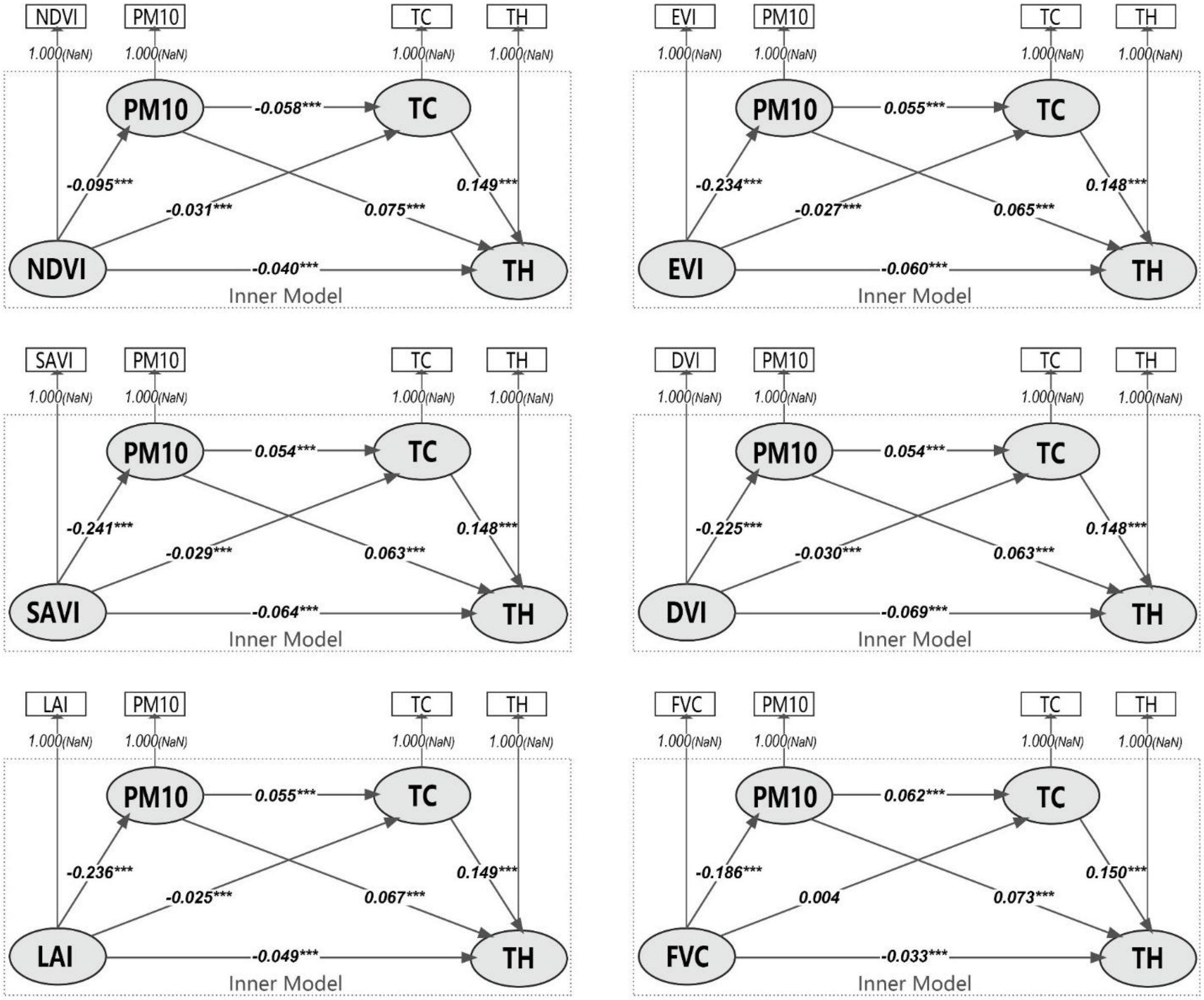
NDVI
PM10
TC
TH
1.000(NaN)
1.000(NaN)
1.000(NaN)
1.000(NaN)
PM10
-0.058***
TC
-0.095***
-0.031***
0.075***
0.149***
NDVI
-0.040***
TH
Inner Model
EVI
PM10
TC
TH
1.000(NaN)
1.000(NaN)
1.000(NaN)
1.000(NaN)
PM10
0.055***
TC
-0.234***
-0.027***
0.065***
0.148***
EVI
-0.060***
TH
Inner Model
SAVI
PM10
TC
TH
1.000(NaN)
1.000(NaN)
1.000(NaN)
1.000(NaN)
PM10
0.054***
TC
-0.241***
-0.029***
0.063***
0.148***
SAVI
-0.064***
TH
Inner Model
DVI
PM10
TC
TH
1.000(NaN)
1.000(NaN)
1.000(NaN)
1.000(NaN)
PM10
0.054***
TC
-0.225***
-0.030***
0.063***
0.148***
DVI
-0.069***
TH
Inner Model
LAI
PM10
TC
TH
1.000(NaN)
1.000(NaN)
1.000(NaN)
1.000(NaN)
PM10
0.055***
TC
-0.236***
-0.025***
0.067***
0.149***
LAI
-0.049***
TH
Inner Model
FVC
PM10
TC
TH
1.000(NaN)
1.000(NaN)
1.000(NaN)
1.000(NaN)
PM10
0.062***
TC
-0.186***
0.004
0.073***
0.150***
FVC
-0.033***
TH
Inner Model

**(b)**

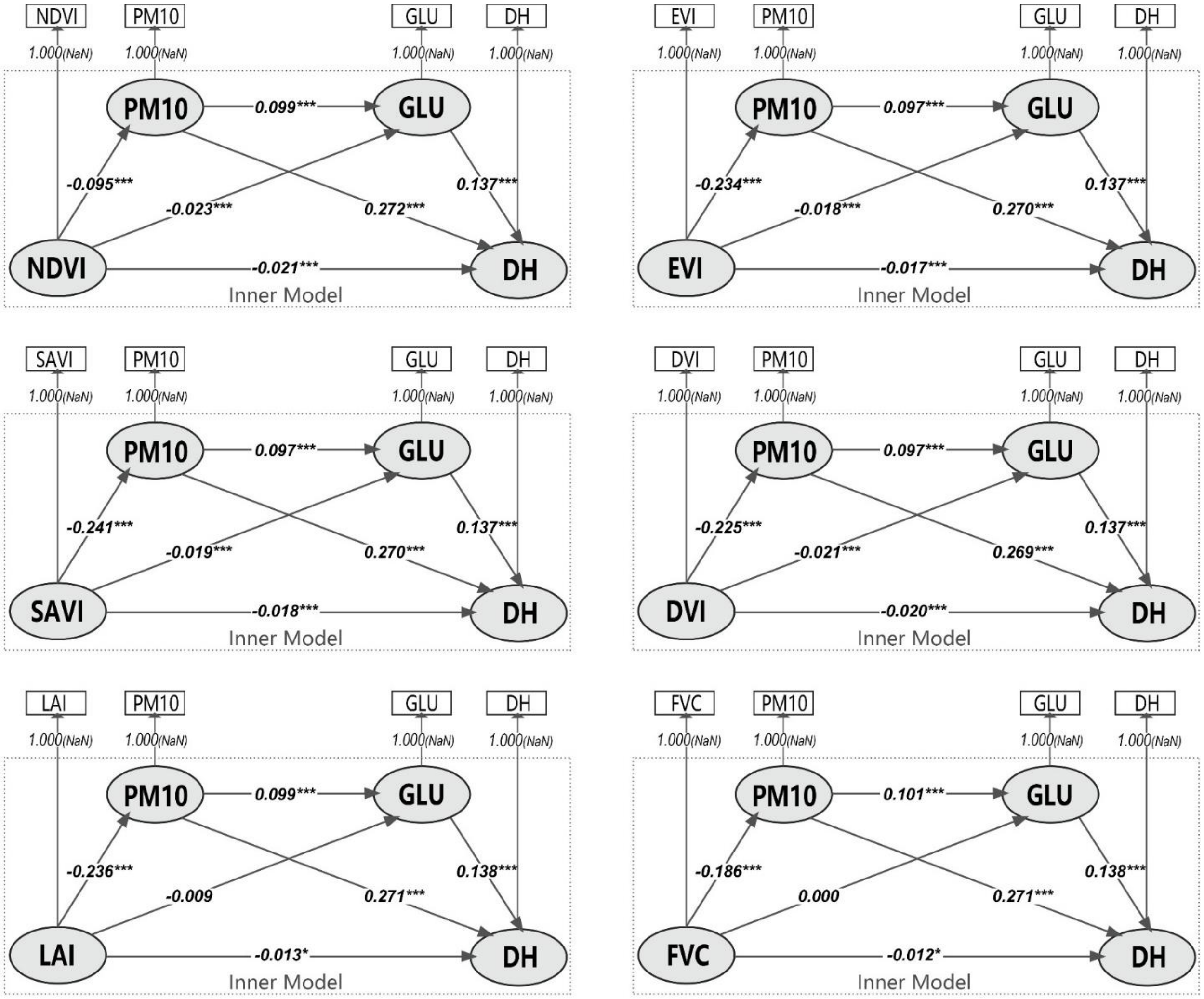
NDVI
PM10
GLU
DH
1.000(NaN)
1.000(NaN)
1.000(NaN)
1.000(NaN)
PM10
GLU
0.099***
-0.095***
-0.023***
0.272***
0.137***
NDVI
-0.021***
DH
Inner Model
EVI
PM10
GLU
DH
1.000(NaN)
1.000(NaN)
1.000(NaN)
1.000(NaN)
PM10
GLU
0.097***
-0.234***
-0.018***
0.270***
0.137***
EVI
-0.017***
DH
Inner Model
SAVI
PM10
GLU
DH
1.000(NaN)
1.000(NaN)
1.000(NaN)
1.000(NaN)
PM10
GLU
0.097***
-0.241***
-0.019***
0.270***
0.137***
SAVI
-0.018***
DH
Inner Model
DVI
PM10
GLU
DH
1.000(NaN)
1.000(NaN)
1.000(NaN)
1.000(NaN)
PM10
GLU
0.097***
-0.225***
-0.021***
0.269***
0.137***
DVI
-0.020***
DH
Inner Model
LAI
PM10
GLU
DH
1.000(NaN)
1.000(NaN)
1.000(NaN)
1.000(NaN)
PM10
GLU
0.099***
-0.236***
-0.009
0.271***
0.138***
LAI
-0.013*
DH
Inner Model
FVC
PM10
GLU
DH
1.000(NaN)
1.000(NaN)
1.000(NaN)
1.000(NaN)
PM10
GLU
0.101***
-0.186***
0.000
0.271***
0.138***
FVC
-0.012*
DH
Inner Model

**(c)**

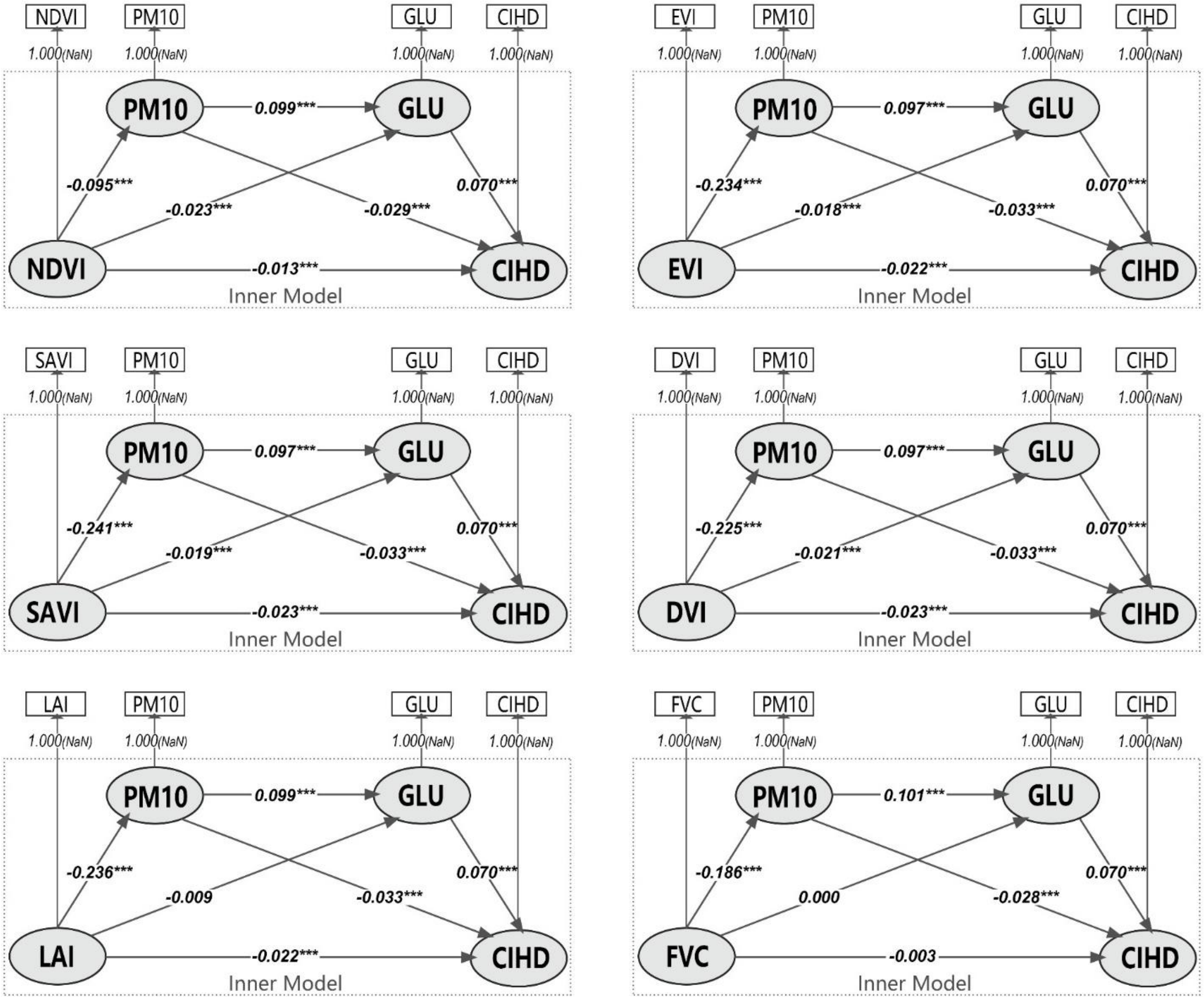
NDVI
PM10
GLU
CIHD
1.000(NaN)
1.000(NaN)
1.000(NaN)
1.000(NaN)
PM10
0.099***
GLU
-0.095***
-0.023***
-0.029***
0.070***
NDVI
-0.013***
CIHD
Inner Model
EVI
PM10
GLU
CIHD
1.000(NaN)
1.000(NaN)
1.000(NaN)
1.000(NaN)
PM10
0.097***
GLU
-0.234***
-0.018***
-0.033***
0.070***
EVI
-0.022***
CIHD
Inner Model
SAVI
PM10
GLU
CIHD
1.000(NaN)
1.000(NaN)
1.000(NaN)
1.000(NaN)
PM10
0.097***
GLU
-0.241***
-0.019***
-0.033***
0.070***
SAVI
-0.023***
CIHD
Inner Model
DVI
PM10
GLU
CIHD
1.000(NaN)
1.000(NaN)
1.000(NaN)
1.000(NaN)
PM10
0.097***
GLU
-0.225***
-0.021***
-0.033***
0.070***
DVI
-0.023***
CIHD
Inner Model
LAI
PM10
GLU
CIHD
1.000(NaN)
1.000(NaN)
1.000(NaN)
1.000(NaN)
PM10
0.099***
GLU
-0.236***
-0.009
-0.033***
0.070***
LAI
-0.022***
CIHD
Inner Model
FVC
PM10
GLU
CIHD
1.000(NaN)
1.000(NaN)
1.000(NaN)
1.000(NaN)
PM10
0.101***
GLU
-0.186***
0.000
-0.028***
0.070***
FVC
-0.003
CIHD
Inner Model

**(d)**

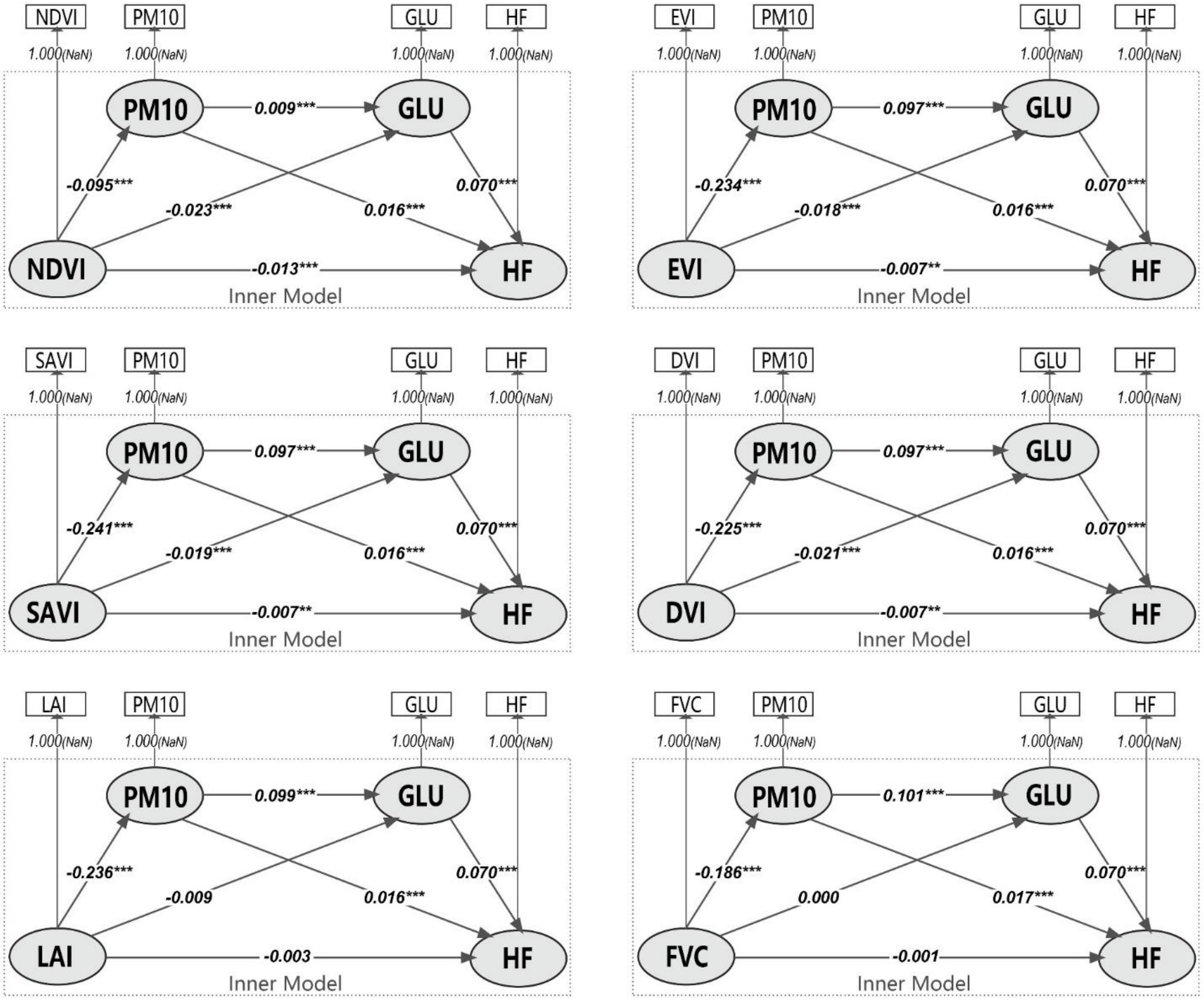


Abbreviation, TH (Times of hospitalizations), DH (Days of hospitalizations), CIHD (Diagnosed chronic ischemic heart disease), HF (Diagnosed heart failure), TC (Total cholesterol), GLU (Blood glucose), NDVI (Normalized difference vegetation index), EVI (Enhanced vegetation index), SAVI (Soil adjusted vegetation index), DVI (Difference vegetation index), FVC (Fractional Vegetation Cover), LAI (Leaf area index).

**Figure S3.** Path diagrams of the single-factor effect PLS-SEMs in Model group 4. (a) TH, (b) DH, (c) CIHD, (d) HF.
